# Supplementary material for: Identification of Freezing-Responsive microRNAs and Their Targets in Chinese Jujube by Small RNA and Degradome Sequencing
Source: Int J Mol Sci. 2026 Sep 4;27(17):7912. doi: 10.3390/ijms27177912 (PMC13566890; doi:10.3390/ijms27177912)
Supplement: Supplementary file 1 [file ijms-27-07912-s001.zip › Supplementary Material S2/novel_miR_123.pdf]

Provisional ID	:	novel_miR_123
Score total	:	106162.9
Score for star read(s)	:	3.9
Score for read counts	:	106154.4
Score for mfe	:	3
Score for randfold	:	1.6
Score for cons. seed	:	
Total read count	:	208229
Mature read count	:	191761
Loop read count	:	9
Star read count	:	16459

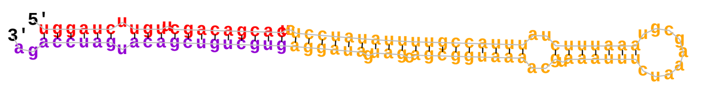

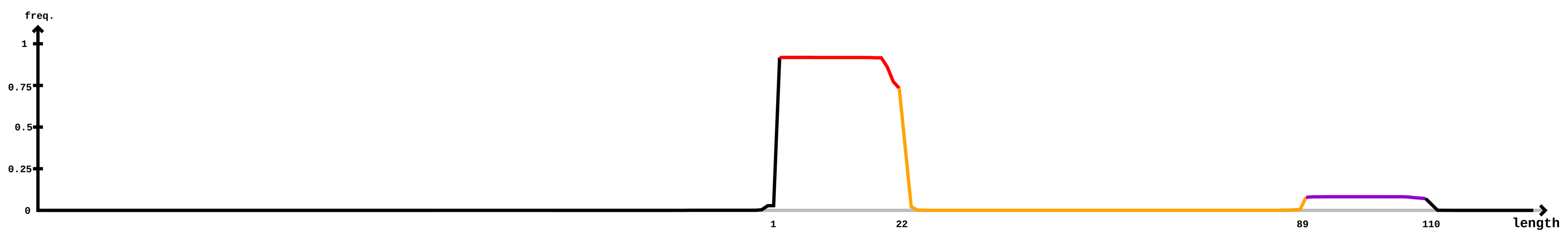


```
                                                                                                                          Mature                                                                                      Star
5'- uguuuucaucaacaguuagguuaaaaagaaaaugagcuccucuuauuuugucaucaaguauaaaaugacggcgagcucuuacagaugaaagcagugcgguuccaucuuguucguuuacccucuggaucuuguucgacagcacucuccuauauuuugccauuuaucuuuaaaugcgaaaucuuuaaaugcaaaauggcgacgaugauaggagugcugucgacaugauccagaaggugcagucgaauaagau -3'  obs
    uguuuucaucaacaguuagguuaaaaagaaaaugagcuccucuuauuuugucaucaaguauaaaaugacggcgagcucuuacagaugaaagcagugcgguuccaucuuguucguuuacccucuggaucuuguucgacagcacucuccuauauuuugccauuuaucuuuaaaugcgaaaucuuuaaaugcaaaauggcgacgaugauaggagugcugucgacaugauccagaaggugcagucgaauaagau       exp
    ((((((((((((......)))..........((((((((.(((((((((........))))))))).)).))))))....))))))))))..........(((((((((((((((.((((((((.(((.(((((((((..((((((((((((((((((..(((((((.........)))))))).)..))))))))).))).))))))))))))))))))).)))))))).)))).)).))))))))))  reads  mm  sample
    .........................................................guauaaaaugacggcgagcucuuacag......................................................................................................................................................................  1  0  LT-2
    ..............................................................aaaugacggcgagcucuuacagaugaa.................................................................................................................................................................  1  0  JCK-1
    ..............................................................aaaugacggcgagcucuuacagaugaa.................................................................................................................................................................  2  0  JCK-3
    ...............................................................aaugacggcgagcucuuacagaug...................................................................................................................................................................  4  0  LCK-3
    .................................................................ugacggcgagcucuuacagaugaaagcagu...........................................................................................................................................................  1  0  JCK-3
    .................................................................ugacggcgagcucuuacagaugaaagcagu...........................................................................................................................................................  1  0  JCK-2
    .................................................................ugacggcgagcucuuacagaugaaagcagu...........................................................................................................................................................  1  0  JCK-1
    ............................................................................................agugcgguuccaucuuguucguu.......................................................................................................................................  1  0  LT-1
    .....................................................................................................ccaucuuguucguuuacc...................................................................................................................................  1  0  LT-1
    .....................................................................................................ccaucuuguucguuuacccucugg.............................................................................................................................  5  0  LCK-3
    .....................................................................................................ccaucuuguucguuuacccucuggauc..........................................................................................................................  1  0  LT-3
    .......................................................................................................aucuuguucguuuacccucugga............................................................................................................................  2  0  JCK-1
    .........................................................................................................cuuguucguuuacccucuggauc..........................................................................................................................  4  0  LT-2
    ..........................................................................................................uuguucguuuaccUucuggauc..........................................................................................................................  2  1  JCK-1
    ..........................................................................................................uuguucguuuaccUucuggauc..........................................................................................................................  6  1  JCK-2
    ..........................................................................................................uuguucguuuaccUucuggauc..........................................................................................................................  2  1  LT-2
    ..........................................................................................................uuguucguuuaccUucuggauc..........................................................................................................................  3  1  JCK-3
    ...........................................................................................................uguucguuuaccUucuggauc..........................................................................................................................  12  1  LT-3
    ...........................................................................................................uguucguuuaccUucuggauc..........................................................................................................................  10  1  JCK-1
    ...........................................................................................................uguucguuuacccucuggauc..........................................................................................................................  1  0  JCK-1
    ...........................................................................................................uguucguuuaccUucuggauc..........................................................................................................................  3  1  LT-2
    ...........................................................................................................uguucguuuaccUucuggauc..........................................................................................................................  12  1  LT-1
    ...........................................................................................................uguucguuuacccucuggauc..........................................................................................................................  1  0  JCK-2
    ...........................................................................................................uguucguuuaccUucuggauc..........................................................................................................................  31  1  JCK-2
    ...........................................................................................................uguucguuuacccucuggauc..........................................................................................................................  2  0  JCK-3
    ...........................................................................................................uguucguuuaccUucuggauc..........................................................................................................................  30  1  JCK-3
    ..............................................................................................................ucguuuacccucuggaucuug.......................................................................................................................  1  0  LT-2
    .................................................................................................................uuuacccucuggaucuuguA.....................................................................................................................  1  1  JCK-1
    .................................................................................................................uuuacccucuggaucuuguuc....................................................................................................................  1  0  JCK-3
    .................................................................................................................uuuacccucuggaucuuguuc....................................................................................................................  1  0  JCK-2
    ......................................................................................................................ccucuggaucuuguucgaca................................................................................................................  1  0  JCK-1
    ......................................................................................................................ccucuggaucuuguucgacag...............................................................................................................  5  0  LT-3
    ......................................................................................................................ccucuggaucuuguucgacagc..............................................................................................................  4  0  JCK-3
```

Mature Star

```
uguuuucaucaacaguuagguuaaaaagaaaaugagcuccucuuauuuugucaucaaguauaaaaugacggcgagcucuuacagaugaaagcagugcgguuccaucuuguucguuuacccucuggaucuuguucgacagcacucuccuauauuuugccauuuaucuuuaaaugcgaaaucuuuaaaugcaaaauggcgacgaugauaggagugcugucgacaugauccagaaggugcagucgaauaagau
......................................................................................................................ccucuggaucuuguucgacagc..............................................................................................................  8  0  JCK-2
......................................................................................................................ccucuggaucuuguucgacagc..............................................................................................................  6  0  JCK-1
......................................................................................................................ccucuggaucuuguucgacagc..............................................................................................................  2  0  LT-2
......................................................................................................................ccucuggaucuuguucgacagc..............................................................................................................  2  0  LT-1
......................................................................................................................ccucuggaucuuguucgacagc..............................................................................................................  2  0  LT-3
......................................................................................................................ccucuggCucuuguucgacagcacu...........................................................................................................  1  1  JCK-2
......................................................................................................................ccucuggaucuuguucgacagcacu...........................................................................................................  1  0  JCK-2
......................................................................................................................ccucuggaucuuguucgacagcacu...........................................................................................................  1  0  JCK-1
......................................................................................................................ccucuggaucuuguucgacagcacu...........................................................................................................  1  0  JCK-3
.......................................................................................................................cucuggaucuuguucgac.................................................................................................................  1  0  LT-3
.......................................................................................................................cucuggaucuuguucgaca................................................................................................................  8  0  LCK-3
.......................................................................................................................cucuggaucuuguucgaca................................................................................................................  6  0  LT-3
.......................................................................................................................cucuggaucuuguucgaca................................................................................................................  2  0  LT-1
.......................................................................................................................cucuggaucuuguucgaca................................................................................................................  6  0  LT-2
.......................................................................................................................cucuggaucuuguucgacagc..............................................................................................................  43  0  JCK-2
.......................................................................................................................cucuggaucuuguucgacagc..............................................................................................................  36  0  JCK-1
.......................................................................................................................cucuggaucuuguucgacagc..............................................................................................................  5  0  LCK-3
.......................................................................................................................cucuggaucuuguucgacagc..............................................................................................................  22  0  LT-3
.......................................................................................................................cucuggaucuuguucgacagc..............................................................................................................  20  0  LT-1
.......................................................................................................................cucuggaucuuguucgacagc..............................................................................................................  34  0  JCK-3
.......................................................................................................................cCcuggaucuuguucgacagc..............................................................................................................  2  1  LT-1
.......................................................................................................................cucuggCucuuguucgacagc..............................................................................................................  1  1  JCK-2
.......................................................................................................................cucuggaucuuguucgacagc..............................................................................................................  8  0  LT-2
.......................................................................................................................cNcuggaucuuguucgacagca.............................................................................................................  1  1  JCK-3
.......................................................................................................................cucuggaucuuguucgCcagca.............................................................................................................  1  1  LT-1
.......................................................................................................................cucuggaucuuguucgacagUa.............................................................................................................  2  1  JCK-2
.......................................................................................................................cucuggaucuuguucgacaNca.............................................................................................................  1  1  JCK-1
.......................................................................................................................Aucuggaucuuguucgacagca.............................................................................................................  2  1  JCK-3
.......................................................................................................................cNcuggaucuuguucgacagca.............................................................................................................  1  1  JCK-2
.......................................................................................................................cucuggaucuuguucgacagca.............................................................................................................  24  0  LCK-3
.......................................................................................................................cucuggaucuuguucgacagca.............................................................................................................  81  0  JCK-1
.......................................................................................................................cucuggaucuuguucgaAagca.............................................................................................................  1  1  JCK-1
.......................................................................................................................cucuggaucuuguucgacagcU.............................................................................................................  1  1  JCK-1
.......................................................................................................................cucuggaucuuguucgacagca.............................................................................................................  103  0  JCK-3
.......................................................................................................................cucuggaucuuNuucgacagca.............................................................................................................  1  1  JCK-1
.......................................................................................................................cucuggaucuuguucgacagcU.............................................................................................................  3  1  JCK-2
.......................................................................................................................cucuggaucuuguucgacagca.............................................................................................................  12  0  LT-3
.......................................................................................................................cucuggaucuuguucgacagca.............................................................................................................  6  0  LT-2
.......................................................................................................................cucuggaucuuguucgacagcG.............................................................................................................  1  1  JCK-1
.......................................................................................................................cucuggaucuuguucgacCgca.............................................................................................................  1  1  JCK-1
.......................................................................................................................cuUuggaucuuguucgacagca.............................................................................................................  1  1  JCK-3
.......................................................................................................................cucuggaucuuguucgacagca.............................................................................................................  137  0  JCK-2
.......................................................................................................................cucuggaucuuguucgacagca.............................................................................................................  19  0  LT-1
.......................................................................................................................cucuggaucuuguucgacagcacu...........................................................................................................  1  0  JCK-3
.......................................................................................................................cucuggaucuuguucgacagcacucu.........................................................................................................  1  0  LT-3
........................................................................................................................ucuggaucuuguucgaca................................................................................................................  14  0  JT-2
........................................................................................................................ucuggaucuuguucgaca................................................................................................................  12  0  JT-1
........................................................................................................................ucuggaucuuguucgacU................................................................................................................  1  1  LT-3
........................................................................................................................ucuggaucuuguucgaca................................................................................................................  23  0  JCK-2
........................................................................................................................ucuggaucuuguucgCca................................................................................................................  1  1  LT-3
........................................................................................................................ucuggaucuuguucgaca................................................................................................................  53  0  LT-3
........................................................................................................................ucuggaucuuguucgacU................................................................................................................  1  1  JCK-2
........................................................................................................................ucuggaucuuguucgaca................................................................................................................  17  0  JT-3
........................................................................................................................ucuggaucuuguucgaca................................................................................................................  17  0  LT-1
........................................................................................................................ucuggaucuuguucgaca................................................................................................................  22  0  JCK-3
........................................................................................................................ucuggaucuuguucgaca................................................................................................................  76  0  LCK-3
........................................................................................................................ucuggaucuuguucgacU................................................................................................................  2  1  LT-1
........................................................................................................................ucuggaucuuguucgaca................................................................................................................  25  0  JCK-1
........................................................................................................................ucuggaucuuguucgaca................................................................................................................  37  0  LT-2
........................................................................................................................ucuggaucuuguucgacU................................................................................................................  2  1  LCK-3
........................................................................................................................ucuggaucuuguucgacU................................................................................................................  1  1  JCK-1
........................................................................................................................ucuggaucuuguucgacaU...............................................................................................................  2  1  JCK-3
........................................................................................................................ucuggaucuuguucgacag...............................................................................................................  1  0  LT-3
........................................................................................................................ucuggaucuuguucgacaU...............................................................................................................  2  1  JCK-2
........................................................................................................................ucuggaucuuguucgacag...............................................................................................................  1  0  JCK-1
........................................................................................................................ucuggaucuuguucgacaA...............................................................................................................  1  1  LT-1
........................................................................................................................ucuggaucuuguucgacagc..............................................................................................................  52  0  LT-1
........................................................................................................................ucuggaucuuguucgacagc..............................................................................................................  56  0  JCK-2
........................................................................................................................ucuggNucuuguucgacagc..............................................................................................................  1  1  LCK-1
........................................................................................................................ucuggaucuuguucgacagc..............................................................................................................  28  0  LCK-2
```

Mature Star

```
uguuuucaucaacaguuagguuaaaaagaaaaugagcuccucuuauuuugucaucaaguauaaaaugacggcgagcucuuacagaugaaagcagugcgguuccaucuuguucguuuacccucuggaucuuguucgacagcacucuccuauauuuugccauuuaucuuuaaaugcgaaaucuuuaaaugcaaaauggcgacgaugauaggagugcugucgacaugauccagaaggugcagucgaauaagau
........................................................................................................................ucuggaucuuguucgacagU.............................................................................................................. 1 1 LT-1
........................................................................................................................ucuggaucuuguucgacagc.............................................................................................................. 20 0 LCK-1
........................................................................................................................ucuggCucuuguucgacagc.............................................................................................................. 1 1 LT-1
........................................................................................................................ucuggaucuuguucgacagc.............................................................................................................. 4 0 JT-3
........................................................................................................................ucuggaucuuguucgacagc.............................................................................................................. 29 0 JCK-3
........................................................................................................................ucuggaucuuguucgacagc.............................................................................................................. 80 0 LT-3
........................................................................................................................ucuggaucuuguucgacagc.............................................................................................................. 69 0 LT-2
........................................................................................................................ucuggaucuuguucgacagc.............................................................................................................. 62 0 LCK-3
........................................................................................................................ucuggaucuuguucgacagc.............................................................................................................. 33 0 JCK-1
........................................................................................................................ucuggaucuuguucgacagc.............................................................................................................. 5 0 JT-2
........................................................................................................................uUuggaucuuguucgacagca............................................................................................................. 5 1 JCK-1
........................................................................................................................ucuggaucuuguucgacagca............................................................................................................. 31 0 LCK-2
........................................................................................................................ucuggauAuuguucgacagca............................................................................................................. 1 1 JCK-3
........................................................................................................................ucuggaucuuguucgacagUa............................................................................................................. 1 1 JCK-3
........................................................................................................................ucuggaucuugNucgacagca............................................................................................................. 2 1 JCK-1
........................................................................................................................ucuggaucuuguucgaAagca............................................................................................................. 1 1 JCK-2
........................................................................................................................ucuggaucCuguucgacagca............................................................................................................. 1 1 JCK-2
........................................................................................................................ucuggaucuuguucgacagca............................................................................................................. 56 0 JT-1
........................................................................................................................uNuggaucuuguucgacagca............................................................................................................. 2 1 JCK-2
........................................................................................................................ucuggaucuuguucgacagca............................................................................................................. 1162 0 JCK-3
........................................................................................................................ucCggaucuuguucgacagca............................................................................................................. 1 1 JCK-3
........................................................................................................................ucuggaucuuguucgCcagca............................................................................................................. 1 1 JCK-1
........................................................................................................................ucNggaucuuguucgacagca............................................................................................................. 1 1 LCK-1
........................................................................................................................ucuggaucuuguucgaAagca............................................................................................................. 1 1 JCK-3
........................................................................................................................ucuggaucuuguucgacagcU............................................................................................................. 3 1 LT-2
........................................................................................................................Ccuggaucuuguucgacagca............................................................................................................. 1 1 LT-3
........................................................................................................................ucuggaucuuguucgacaUca............................................................................................................. 3 1 JCK-2
........................................................................................................................ucuggaucCuguucgacagca............................................................................................................. 1 1 LT-2
........................................................................................................................ucuggaucuuguucgacaAca............................................................................................................. 1 1 JCK-2
........................................................................................................................ucuggaucuuguucgacagca............................................................................................................. 19 0 JT-3
........................................................................................................................ucuggaucuuguucgacagcU............................................................................................................. 3 1 JCK-3
........................................................................................................................uUuggaucuuguucgacagca............................................................................................................. 5 1 JCK-2
........................................................................................................................ucuggaucuuguucgacagca............................................................................................................. 128 0 LT-3
........................................................................................................................ucuggaucuuguucgacagca............................................................................................................. 76 0 LCK-3
........................................................................................................................ucuggaucuuguucgaUagca............................................................................................................. 1 1 JCK-3
........................................................................................................................Ccuggaucuuguucgacagca............................................................................................................. 1 1 JCK-2
........................................................................................................................ucuggaucuuguucgacaUca............................................................................................................. 1 1 JCK-1
........................................................................................................................ucuggCucuuguucgacagca............................................................................................................. 1 1 JCK-3
........................................................................................................................ucuggaucuuguucgacagcU............................................................................................................. 2 1 LT-3
........................................................................................................................ucuggaucuuguucgaAagca............................................................................................................. 1 1 JCK-1
........................................................................................................................ucuggaucuuguucgCcagca............................................................................................................. 1 1 LT-1
........................................................................................................................ucuggaucuuguucgacagcU............................................................................................................. 8 1 JCK-2
........................................................................................................................ucuggaucuuguucgacagcU............................................................................................................. 2 1 JCK-1
........................................................................................................................ucuggaucuuguucgacagca............................................................................................................. 83 0 LT-2
........................................................................................................................ucuggaucuuguucAacagca............................................................................................................. 1 1 JCK-1
........................................................................................................................ucuggaucuuguucgacagca............................................................................................................. 1463 0 JCK-2
........................................................................................................................ucuggaucuuguucgacagca............................................................................................................. 27 0 LCK-1
........................................................................................................................ucugAaucuuguucgacagca............................................................................................................. 1 1 JCK-1
........................................................................................................................ucuggaucuuguucgacagcU............................................................................................................. 3 1 LCK-3
........................................................................................................................ucuggaucuuguucgCcagca............................................................................................................. 1 1 JT-1
........................................................................................................................ucuggCucuuguucgacagca............................................................................................................. 4 1 JCK-2
........................................................................................................................uUuggaucuuguucgacagca............................................................................................................. 5 1 JCK-3
........................................................................................................................ucuggaucuuguucgacagca............................................................................................................. 9 0 JT-2
........................................................................................................................ucuggaucuuguucgacagcN............................................................................................................. 1 1 JCK-1
........................................................................................................................ucuggaucuuguucgacagca............................................................................................................. 882 0 JCK-1
........................................................................................................................ucuggaucuuguucgacagcC............................................................................................................. 1 1 JCK-1
........................................................................................................................ucuggaucuuguucgacagca............................................................................................................. 109 0 LT-1
........................................................................................................................ucuggaucuuguucgacagcac............................................................................................................ 30 0 JCK-3
........................................................................................................................ucuggaucuuguucgacagcac............................................................................................................ 10 0 LT-1
........................................................................................................................ucuggaucuuguucgacagcac............................................................................................................ 16 0 LCK-3
........................................................................................................................ucuggaucuuguucgacagcac............................................................................................................ 7 0 LT-2
........................................................................................................................ucuggaucuuguucgacagcac............................................................................................................ 16 0 JCK-1
........................................................................................................................ucuggaucuuguucgacagcac............................................................................................................ 11 0 LT-3
........................................................................................................................ucuggaucuuguucgacagcaA............................................................................................................ 1 1 JCK-3
........................................................................................................................ucuggaucuuguucgacagcac............................................................................................................ 37 0 JCK-2
........................................................................................................................ucuggaucuuguucgacagcacu........................................................................................................... 9 0 JCK-2
........................................................................................................................ucuggaucuuguucgacagcacC........................................................................................................... 1 1 LT-3
........................................................................................................................ucuggaucuuguucgacagcacu........................................................................................................... 2 0 LT-2
........................................................................................................................ucuggaucuuguucgacagcacu........................................................................................................... 5 0 JCK-3
........................................................................................................................ucuggaucuuguucgacagcacA........................................................................................................... 2 1 JCK-1
```

```
                                                                                                                          Mature                                                                             Star
uguuuucaucaacaguuagguuaaaaagaaaaugagcuccucuuauuuugucaucaaguauaaaaugacggcgagcucuuacagaugaaagcagugcgguuccaucuuguucguuuacccucuggaucuuguucgacagcacucuccuauauuuugccauuuaucuuuaaaugcgaaaucuuuaaaugcaaaauggcgacgaugauaggagugcugucgacaugauccagaaggugcagucgaauaagau
........................................................................................................................ucuggaucuuguucgacagcaUu...........................................................................................................    1     1    LT-3
........................................................................................................................ucuggaucuuguucgacagcaAu...........................................................................................................    3     1    LT-3
........................................................................................................................ucuggaucuuguucgacagcacA...........................................................................................................    1     1    JCK-2
........................................................................................................................ucuggaucuuguucgacagcacu...........................................................................................................    8     0    LCK-3
........................................................................................................................ucuggaucuuguucgacagcacu...........................................................................................................    4     0    JCK-1
........................................................................................................................ucuggaucuuguucgacagcacA...........................................................................................................    1     1    JCK-3
........................................................................................................................ucuggaucuuguucgacagcacu...........................................................................................................    3     0    LT-3
........................................................................................................................ucuggaucuuguucgacagcacuc..........................................................................................................    1     0    JCK-3
........................................................................................................................ucuggaucuuguucgacagcacuc..........................................................................................................    3     0    LT-1
........................................................................................................................ucuggaucuuguucgacagcacuc..........................................................................................................    5     0    JCK-1
........................................................................................................................ucuggaucuuguucgacagcacuc..........................................................................................................    1     0    LT-3
........................................................................................................................ucuggaucuuguucgacagcacuU..........................................................................................................    1     1    LT-1
........................................................................................................................ucuggaucuuguucgacagcacuc..........................................................................................................    3     0    JCK-2
........................................................................................................................ucuggaucuuguucgacagcacucu.........................................................................................................    1     0    LT-1
........................................................................................................................ucuggaucuuguucgacagcacucu.........................................................................................................    2     0    JCK-2
.........................................................................................................................cuggaucuuguucgacagc..............................................................................................................    1     0    LCK-3
.........................................................................................................................cuggaucuuguucgacagc..............................................................................................................    1     0    LT-1
.........................................................................................................................cuggaucuuguucgacagc..............................................................................................................    4     0    LT-3
.........................................................................................................................cuggaucuuguucgacagc..............................................................................................................    3     0    LT-2
.........................................................................................................................cuggaucuuguucgacagca.............................................................................................................    4     0    LT-3
.........................................................................................................................cuggaucuuguucgacagca.............................................................................................................    2     0    LT-1
.........................................................................................................................cuggaucuuguucgacagca.............................................................................................................    12    0    JCK-3
.........................................................................................................................cuggaucuuguucgacagca.............................................................................................................    7     0    LCK-3
.........................................................................................................................cuggaucuuguucgacagca.............................................................................................................    17    0    JCK-2
.........................................................................................................................cuggaucuuguucgacagcU.............................................................................................................    1     1    JCK-3
.........................................................................................................................cuggaucuuguucgacagca.............................................................................................................    15    0    JCK-1
.........................................................................................................................cuggaucAuguucgacagca.............................................................................................................    1     1    LT-1
.........................................................................................................................cuggaucuuguucgacagcac............................................................................................................    4     0    LT-2
.........................................................................................................................cuggaucuuguucgacagcac............................................................................................................    1     0    JCK-3
.........................................................................................................................cuggaucuuguucgacagcac............................................................................................................    2     0    JCK-2
.........................................................................................................................cuggaucuuguucgacagcacu...........................................................................................................    15    0    JCK-1
.........................................................................................................................cuggaucuuguucgacagcacu...........................................................................................................    27    0    JCK-2
.........................................................................................................................cuggaucuuguucgacagcacu...........................................................................................................    13    0    JCK-3
.........................................................................................................................cuggaucuuguucgacagcacu...........................................................................................................    2     0    LT-1
.........................................................................................................................cuggaucuuguucgacagcacu...........................................................................................................    3     0    LT-2
.........................................................................................................................cuggaucuuguucgacagcacu...........................................................................................................    5     0    LCK-3
.........................................................................................................................cuggaucuuguucgacagcacu...........................................................................................................    2     0    LT-3
.........................................................................................................................Uuggaucuuguucgacagcacu...........................................................................................................    1     1    JCK-3
.........................................................................................................................Uuggaucuuguucgacagcacuc..........................................................................................................    1     1    JCK-2
.........................................................................................................................cuggaucuuguucgacagcacuc..........................................................................................................    1     0    JCK-1
.........................................................................................................................cuggaucuuguucgacagcacuc..........................................................................................................    6     0    JCK-2
.........................................................................................................................cuggaucuuguucgacagcacuc..........................................................................................................    1     0    JCK-3
.........................................................................................................................cuggaucuuguucgacagcacuc..........................................................................................................    2     0    LT-3
..........................................................................................................................uggaucuuguucgacagc..............................................................................................................    454   0    JCK-1
..........................................................................................................................uggaucuuguucgacagU..............................................................................................................    15    1    LT-3
..........................................................................................................................uAgaucuuguucgacagc..............................................................................................................    1     1    LT-2
..........................................................................................................................uggaucuuguucgCcagc..............................................................................................................    1     1    LT-3
..........................................................................................................................uggaucuuguucgCcagc..............................................................................................................    2     1    LCK-2
..........................................................................................................................uggCucuuguucgacagc..............................................................................................................    3     1    JCK-3
..........................................................................................................................uggaucuuguucgaAagc..............................................................................................................    1     1    LCK-3
..........................................................................................................................uggaucuuguuNgacagc..............................................................................................................    1     1    JT-1
..........................................................................................................................uUgaucuuguucgacagc..............................................................................................................    1     1    LT-3
..........................................................................................................................uggaucuuguucgacagA..............................................................................................................    1     1    LT-3
..........................................................................................................................uggaucuuguucgCcagc..............................................................................................................    4     1    LCK-3
..........................................................................................................................uggaucuuguucgNcagc..............................................................................................................    2     1    LCK-1
..........................................................................................................................ugNaucuuguucgacagc..............................................................................................................    4     1    LT-2
..........................................................................................................................uggaucuuguucgacaAc..............................................................................................................    1     1    LT-2
..........................................................................................................................uggaucuuguucgaUagc..............................................................................................................    1     1    LCK-3
..........................................................................................................................uggaucuuguucgGcagc..............................................................................................................    1     1    LCK-3
..........................................................................................................................uggCucuuguucgacagc..............................................................................................................    3     1    LT-3
..........................................................................................................................uggaucuuguucgaUagc..............................................................................................................    1     1    LT-2
..........................................................................................................................uggaucuuguucgacagU..............................................................................................................    1     1    JCK-1
..........................................................................................................................uggCucuuguucgacagc..............................................................................................................    6     1    LCK-3
..........................................................................................................................uggaucuuguucgaUagc..............................................................................................................    1     1    JCK-1
..........................................................................................................................uggaucuuguucgacaAc..............................................................................................................    1     1    LT-1
..........................................................................................................................uggaucuuguucgacagc..............................................................................................................    659   0    LCK-1
..........................................................................................................................uggaucuuguucgaAagc..............................................................................................................    1     1    LT-2
..........................................................................................................................uggaucuuguucgCcagc..............................................................................................................    2     1    JCK-3
..........................................................................................................................ugAaucuuguucgacagc..............................................................................................................    2     1    JCK-2
..........................................................................................................................uggCucuuguucgacagc..............................................................................................................    19    1    LT-2
```

Mature | Star

uguuuucaucaacaguuagguuaaaaagaaaaugagcuccucuuauuuugucaucaaguauaaaaugacggcgagcucuuacagaugaaagcagugcgguuccaucuuguucguuuacccucuggaucuuguucgacagcacucuccuauauuuugccauuuaucuuuaaaugcgaaaucuuuaaaugcaaaauggcgacgaugauaggagugcugucgacaugauccagaaggugcagucgaauaagau

uggaucuuguucgacagc	794	0	JCK-2
uggaucuuguucgacagc	81	0	JT-1
uAgaucuuguucgacagc	1	1	LCK-1
uggGucuuguucgacagc	2	1	JCK-1
uggaucuuguucgCcagc	8	1	LT-2
uggaucuuguucgacagc	74	0	JT-3
ugAaucuuguucgacagc	1	1	JCK-3
uggaucuuguucgacagU	5	1	LCK-3
uggCucuuguucgacagc	1	1	JCK-1
uggaucuuguucgacaUc	1	1	JCK-1
uggaucuuguucgacagc	616	0	JCK-3
uggaucuuguucgacGgc	1	1	JCK-2
uggaucuuguucgacagU	1	1	LT-2
ugAaucuuguucgacagc	1	1	LCK-3
uggaucuuguucAacagc	1	1	LT-2
ugNaucuuguucgacagc	1	1	LCK-3
ugNaucuuguucgacagc	1	1	LCK-1
Cggaucuuguucgacagc	1	1	LT-2
uggaucuuguucgacCgc	1	1	LT-2
uggaucuuguucgacagU	3	1	LT-1
uNgaucuuguucgacagc	1	1	LCK-1
uggaucuuguucgacagc	2025	0	LT-2
ugAaucuuguucgacagc	1	1	LCK-1
uAgaucuuguucgacagc	2	1	LCK-3
ugUaucuuguucgacagc	1	1	LT-3
uggaucuuguucgacaUc	6	1	LT-2
uggaucuuguucgacaAc	1	1	LCK-3
uNgaucuuguucgacagc	1	1	JT-1
uAgaucuuguucgacagc	2	1	JCK-2
uggaucuuguuNgacagc	1	1	JCK-1
uggaucuuguucgCcagc	3	1	JCK-2
uggaucuuguucUacagc	1	1	JCK-3
uggaucuuguucgacaUc	2	1	JCK-3
uNgaucuuguucgacagc	1	1	JCK-2
uggCucuuguucgacagc	3	1	LT-1
uggaucuuguuNgacagc	1	1	JCK-2
uggaucuuguucgCcagc	2	1	LT-1
uggaucuuguucgacagc	663	0	LCK-2
uggaucuuguucgacCgc	1	1	JCK-3
uggCucuuguucgacagc	1	1	LCK-2
ugAaucuuguucgacagc	2	1	LT-2
uggCucuuguucgacagc	1	1	LCK-1
uggaucuuguucgacagU	3	1	JCK-3
uggaucuuguucgacagc	1173	0	LT-3
uggaucuuguucgacagU	2	1	JCK-2
uggaCcuuguucgacagc	4	1	LCK-3
uggaucuuguucgacagc	48	0	JT-2
uNgaucuuguucgacagc	1	1	LT-3
uggaucuuguucgacagc	3342	0	LCK-3
uggaucuuguucgCcagc	1	1	LCK-1
uggGucuuguucgacagc	1	1	JT-1
uggaucuuguucgacagc	890	0	LT-1
uggaucuuguucgacagca	1963	0	LCK-2
ugCaucuuguucgacagca	1	1	LT-2
uggaucuuguucgCcagca	1	1	LCK-2
uggCucuuguucgacagca	1	1	JCK-2
uggaucuuguucgCcagca	3	1	LT-2
uggaucuuguucgacagca	1924	0	LCK-1
uggaucuuguucgacagUa	1	1	JCK-1
uggaucuuguucgacagGa	1	1	JCK-2
uggauUuuguucgacagca	1	1	LCK-3
uggaucuuguucgCcagca	2	1	JCK-3
uggaucCuguucgacagca	1	1	JCK-1
uggaucuuguucgacagca	932	0	JCK-2
uggaucuuguucgaAagca	3	1	LCK-2
Cggaucuuguucgacagca	1	1	LCK-2
uggaucuuguucgacagcC	1	1	LCK-3
uggaucuuguucgacagcU	9	1	JT-2
uggaucCuguucgacagca	1	1	LCK-1
uggaucuuguucgCcagca	2	1	LT-1

```
                                                                                                                          Mature                                                                                Star
uguuuucaucaacaguuagguuaaaaagaaaaugagcuccucuuauuuugucaucaaguauaaaaugacggcgagcucuuacagaugaaagcagugcgguuccaucuuguucguuuacccucuggaucuuguucgacagcacucuccuauauuuugccauuuaucuuuaaaugcgaaaucuuuaaaugcaaaauggcgacgaugauaggagugcugucgacaugauccagaaggugcagucgaauaagau
..........................................................................................................................uggaucuuguucgaUagca.............................................................................................................  2     1  LCK-2
..........................................................................................................................uggaucuuguucgacagcU.............................................................................................................  68    1  LCK-1
..........................................................................................................................Cggaucuuguucgacagca.............................................................................................................  1     1  LT-2
..........................................................................................................................uggaucuuguucgacagcU.............................................................................................................  56    1  JT-1
..........................................................................................................................uggCucuuguucgacagca.............................................................................................................  1     1  JCK-1
..........................................................................................................................ugAaucuuguucgacagca.............................................................................................................  1     1  JCK-2
..........................................................................................................................uggaCcuuguucgacagca.............................................................................................................  2     1  JCK-3
..........................................................................................................................uggaucuuguucgaUagca.............................................................................................................  4     1  JCK-1
..........................................................................................................................uggaucuuguucgacGgca.............................................................................................................  1     1  JT-1
..........................................................................................................................uggaucuuAuucgacagca.............................................................................................................  1     1  LCK-1
..........................................................................................................................uggauNuuguucgacagca.............................................................................................................  3     1  LCK-1
..........................................................................................................................uNgaucuuguucgacagca.............................................................................................................  3     1  JT-1
..........................................................................................................................uggaucuuguucAacagca.............................................................................................................  1     1  LT-3
..........................................................................................................................uggauUuuguucgacagca.............................................................................................................  1     1  JCK-3
..........................................................................................................................uggaucuuguucgacagUa.............................................................................................................  9     1  LCK-3
..........................................................................................................................uggaucCuguucgacagca.............................................................................................................  1     1  JT-1
..........................................................................................................................uggCucuuguucgacagca.............................................................................................................  2     1  LCK-3
..........................................................................................................................uggNucuuguucgacagca.............................................................................................................  1     1  JCK-1
..........................................................................................................................uUgaucuuguucgacagca.............................................................................................................  1     1  LT-3
..........................................................................................................................uggaucuuguucgacagca.............................................................................................................  237   0  JT-2
..........................................................................................................................uUgaucuuguucgacagca.............................................................................................................  1     1  LCK-2
..........................................................................................................................uggaucuuguucgacagcC.............................................................................................................  1     1  LCK-2
..........................................................................................................................uNgaucuuguucgacagca.............................................................................................................  2     1  JCK-3
..........................................................................................................................uggaucuuguucgacagUa.............................................................................................................  6     1  JCK-2
..........................................................................................................................uggaucuuguucgacagGa.............................................................................................................  1     1  JCK-1
..........................................................................................................................uggaucuuguucgaAagca.............................................................................................................  1     1  LT-2
..........................................................................................................................uggaucuuguucgacagcU.............................................................................................................  211   1  LT-2
..........................................................................................................................uggaucuuguucgacagcU.............................................................................................................  299   1  LCK-3
..........................................................................................................................uggaucuuguucgacaAca.............................................................................................................  1     1  JT-1
..........................................................................................................................uggaucuuguucgNcagca.............................................................................................................  6     1  LCK-1
..........................................................................................................................uggCucuuguucgacagca.............................................................................................................  1     1  JCK-3
..........................................................................................................................uggCucuuguucgacagca.............................................................................................................  5     1  LT-2
..........................................................................................................................uAgaucuuguucgacagca.............................................................................................................  1     1  LT-2
..........................................................................................................................uggaucuuguucgCcagca.............................................................................................................  1     1  LCK-3
..........................................................................................................................uggaucuuguucgacagcU.............................................................................................................  62    1  LCK-2
..........................................................................................................................uggaucuuguucgCcagca.............................................................................................................  5     1  LCK-1
..........................................................................................................................uggaucuuguucgacagcG.............................................................................................................  3     1  JCK-2
..........................................................................................................................uggCucuuguucgacagca.............................................................................................................  8     1  LCK-2
..........................................................................................................................uggaucuuguucgacCgca.............................................................................................................  2     1  LCK-2
..........................................................................................................................uggaucuuguucgacagcC.............................................................................................................  2     1  LT-3
..........................................................................................................................uggaucuuguucgacagcC.............................................................................................................  1     1  JCK-2
..........................................................................................................................uggaucuuguucgaAagca.............................................................................................................  1     1  LT-3
..........................................................................................................................uggaucuuguucgacagcC.............................................................................................................  1     1  JT-1
..........................................................................................................................uggaucuuguucgacCgca.............................................................................................................  2     1  LT-2
..........................................................................................................................uggaucuuguucgCcagca.............................................................................................................  1     1  JT-1
..........................................................................................................................uggaucuuguucgacagca.............................................................................................................  575   0  JT-1
..........................................................................................................................uggGucuuguucgacagca.............................................................................................................  1     1  LCK-3
..........................................................................................................................uggaucuuguucgacCgca.............................................................................................................  1     1  LT-3
..........................................................................................................................uggaucuuguucgacagca.............................................................................................................  1113  0  LT-3
..........................................................................................................................ugAaucuuguucgacagca.............................................................................................................  1     1  LT-2
..........................................................................................................................uggaucuuguucgacCgca.............................................................................................................  1     1  LCK-3
..........................................................................................................................uggaucuuguucgaUagca.............................................................................................................  1     1  JCK-3
..........................................................................................................................uAgaucuuguucgacagca.............................................................................................................  2     1  LCK-3
..........................................................................................................................uggaucuuguucgacagca.............................................................................................................  2250  0  LCK-3
..........................................................................................................................uggaucuuguucNacagca.............................................................................................................  1     1  LCK-1
..........................................................................................................................uAgaucuuguucgacagca.............................................................................................................  1     1  JCK-2
..........................................................................................................................uggGucuuguucgacagca.............................................................................................................  1     1  LCK-2
..........................................................................................................................uggaucuuguucgacagcU.............................................................................................................  194   1  JCK-2
..........................................................................................................................uggaucuuguucgacagca.............................................................................................................  496   0  LT-1
..........................................................................................................................uggaucuuguuGgacagca.............................................................................................................  9     1  LCK-3
..........................................................................................................................uggaucuuguucgacagca.............................................................................................................  529   0  JCK-1
..........................................................................................................................uggaucuuguuNgacagca.............................................................................................................  2     1  JT-1
..........................................................................................................................uggaucuuguucgacagUa.............................................................................................................  10    1  JT-1
..........................................................................................................................uggaucuuguucgacagca.............................................................................................................  1184  0  LT-2
..........................................................................................................................uggaucuuguucgacCgca.............................................................................................................  2     1  JCK-2
..........................................................................................................................uggaucuuguucgacaUca.............................................................................................................  15    1  LCK-3
..........................................................................................................................uNgaucuuguucgacagca.............................................................................................................  3     1  JCK-2
..........................................................................................................................uggaucuuguucgacagAa.............................................................................................................  1     1  LT-3
..........................................................................................................................uggaucCuguucgacagca.............................................................................................................  1     1  LT-3
..........................................................................................................................uggaucuuguucgaUagca.............................................................................................................  4     1  JCK-2
```

Mature | Star

uguuuucaucaacaguuagguuaaaaagaaaaugagcuccucuuauuuugucaucaaguauaaaaugacggcgagcucuuacagaugaaagcagugcgguuccaucuuguucguuuacccucuggaucuuguucgacagcacucuccuauauuuugccauuuaucuuuaaaugcgaaaucuuuaaaugcaaaauggcgacgaugauaggagugcugucgacaugauccagaaggugcagucgaauaagau			
uggaucuuguucgacagcU	164	1	LT-3
ugNaucuuguucgacagca	5	1	LT-2
uggaucuuguucgacagUa	11	1	LT-2
uggaucuuguucgacagcU	155	1	JCK-1
uggaucuuguucgaGagca	1	1	LCK-1
uggaucuuguucgacaUca	3	1	JCK-2
uggaucuuguucgacagUa	4	1	JCK-3
uggaucuuguucgaNagca	4	1	LCK-1
uggaucuuguucgacagcU	75	1	LT-1
uggaucuuguucgacagcU	162	1	JCK-3
uggaucuuguuNgacagca	1	1	JT-2
uggGucuuguucgacagca	1	1	JT-2
uggaucuuguucgacaUca	1	1	LT-3
uggaucuuguucgacaAca	1	1	LT-1
uggaucuuguucgacaAca	1	1	LCK-1
uggaucuuguucgacagca	606	0	JCK-3
uggaucuuguucgacagUa	5	1	LT-1
uggGucuuguucgacagca	1	1	JT-1
uggaucuuguucgacagcU	5	1	JT-3
uggCucuuguucgacagca	5	1	LT-1
uggaucuuguucgacagca	294	0	JT-3
uggCucuuguucgacagca	1	1	LT-3
uggCucuuguucgacagca	2	1	LCK-1
uggaucuuguucgacagUa	5	1	LT-3
uggaucuuguuNgacagca	5	1	JCK-1
uNgaucuuguucgacagca	1	1	LCK-1
uggaucuuguucgacaUca	1	1	JCK-3
uggaucuuguucgaUagca	1	1	LT-2
uggaucuuguucgacagcG	2	1	LT-3
uggaucuuguucgacagcNc	1	1	LCK-1
uggaucuuguucgacagcac	727	0	JCK-2
uggaucuuguucgGcagcac	1	1	LCK-1
uggaucuuguucgacagcNc	1	1	JCK-1
uggaucuuguucgNcagcac	4	1	LCK-1
uggaucuuguucgacagcaU	16	1	LT-3
uggaucuuguuNgacagcac	1	1	JT-2
uggaucuuguucgacagcaU	1	1	LCK-3
uggaucuuguucgacCgcac	2	1	JCK-1
uAgaucuuguucgacagcac	1	1	LT-3
uggaucuuguucgacagcCc	1	1	LCK-2
uggaucuuguucgacagcac	508	0	JCK-3
uggaucuuguucgacagcUc	3	1	LT-2
ugAaucuuguucgacagcac	1	1	LCK-3
uggaucuuguucgacagcCc	1	1	LT-1
uggaucuuguucgacagcaA	2	1	LT-3
uggCucuuguucgacagcac	2	1	JCK-2
uggaucuuguucgaAagcac	1	1	JCK-1
uggaucuuguucgacagcGc	1	1	LT-2
Cggaucuuguucgacagcac	2	1	LT-2
uggaucuuguucgacagcac	762	0	LT-3
uggaucuuguucgacagcac	2109	0	LCK-3
uggaucuuguucgacGgcac	1	1	LT-2
uggaucuuguucgacagcac	821	0	LCK-1
uggaucuuguucgacGgcac	1	1	JCK-2
uggaucuuguucgaAagcac	2	1	LCK-2
ugUaucuuguucgacagcac	1	1	LT-3
uggCucuuguucgacagcac	2	1	LT-3
uggaucuuguucgacagcUc	1	1	JCK-1
uggaucuuguucUacagcac	1	1	LT-1
uggaucuuguucgacagcaU	12	1	JCK-3
uggaucuuguucgCcagcac	5	1	LT-2
uggaCcuuguucgacagcac	1	1	LCK-3
ugUaucuuguucgacagcac	1	1	LT-2
uggaucuuguucgGcagcac	1	1	JCK-1
uggaucuuguucgacagcaU	5	1	JCK-1
uggaucuuguucgacagcUc	2	1	JCK-3
uggauNuuguucgacagcac	2	1	LCK-1
uggaucuugCucgacagcac	1	1	LT-2
uggaucuuguucgacagUac	1	1	JCK-3
uAgaucuuguucgacagcac	1	1	LCK-3

Mature Star

uguuuucaucaacaguuagguuaaaaagaaaaugagcuccucuuauuuugucaucaaguauaaaaugacggcgagcucuuacagaugaaagcagugcgguuccaucuuguucguuuacccucuggaucuuguucgacagcacucuccuauauuuugccauuuaucuuuaaaugcgaaaucuuuaaaugcaaaauggcgacgaugauaggagugcugucgacaugauccagaaggugcagucgaauaagau

uggaucuuguucgacGgcac	1	1	LT-1
uggaucuuguucgCcagcac	6	1	LCK-3
ugAaucuuguucgacagcac	1	1	LT-2
uggaucuuguucgacagcaA	1	1	JCK-1
uggaucuuguucgaAagcac	1	1	LT-3
uggaucuuguucgacagcac	83	0	JT-3
uggaucuuguucgacagcUc	3	1	LT-3
uggaucuuguucgacagcac	72	0	JT-2
uggaucuuguucgacaAcac	1	1	JCK-1
uggaucuuAuucgacagcac	1	1	LCK-1
uggCucuuguucgacagcac	1	1	JT-3
uNgaucuuguucgacagcac	1	1	JCK-1
uggaucuuguucgaUagcac	1	1	LT-2
uggaucuuguucgCcagcac	1	1	LCK-2
uggaucuuguucgCcagcac	1	1	LT-3
uggaucuuguucgacagcCc	1	1	LCK-3
uggaucuuguucgacagcUc	2	1	JCK-2
uggaucuuguucgaUagcac	3	1	JCK-2
uggaucuuguucgacagcac	164	0	JT-1
uggCucuuguucgacagcac	11	1	LT-2
uggaucuuguucgacagcac	454	0	JCK-1
uggaucuuguucAacagcac	1	1	LT-2
uggaucuuguucgCcagcac	1	1	JCK-1
ugNaucuuguucgacagcac	4	1	LT-2
uggaucuuguucgacagcaA	9	1	JCK-3
uggCucuuguucgacagcac	2	1	LCK-1
uggaucuuguucgaUagcac	3	1	JCK-3
uggaucuuguucgaNagcac	4	1	LCK-1
uggaucuuguucgacagcCc	3	1	LT-2
uggaucuuguucgaUagcac	1	1	JCK-1
uggCucuuguucgacagcac	4	1	LCK-2
uggaucuuguucgacagcUc	1	1	LT-1
uggaucuuguucgacagcaU	11	1	LT-2
ugUaucuuguucgacagcac	1	1	JCK-3
uggCucuuguucgacagcac	5	1	LCK-3
uggaucuuguucgacagcaA	3	1	JCK-2
uggaucuuguucgacagcaU	10	1	LT-1
uggaucuuguucgaGagcac	1	1	LCK-1
ugAaucuuguucgacagcac	2	1	JCK-1
uggaucuuguucgacagcac	796	0	LCK-2
uggaucuuguucgacagcac	1028	0	LT-2
uggCucuuguucgacagcac	1	1	LT-1
uggaucuuguucgacagcaU	19	1	JCK-2
uggaucuuguucgacagcac	564	0	LT-1
uggaucuuguucgacCgcac	1	1	LT-2
uggaucuuguucgacagcCc	1	1	JT-3
uggaucuuguucgacagcacG	2	1	LT-3
uggaucuuguucgacGgcacu	1	1	LT-2
uggaucuuguuAgacagcacu	1	1	JCK-1
uAgaucuuguucgacagcacu	1	1	LT-1
uggaucuuguucgCcagcacu	80	1	JCK-2
uggaucuCguucgacagcacu	1	1	LCK-2
uggaucuuguucCacagcacu	1	1	JCK-2
uggaucuuguucgacagcaNu	23	1	JCK-1
ugNaucuuguucgacagcacu	2	1	LCK-1
uggCucuuguucgacagcacu	1	1	JT-2
uggaucuGguucgacagcacu	6	1	JCK-2
uggaucuuguucgacagcCcu	1	1	JT-1
uggaucuuguucgacagcCcu	1	1	LCK-2
uggaucuuguucgacagcacA	61	1	LCK-3
uggaucuuguucgacCgcacu	10	1	JCK-3
uggaucuuguucgacagcacC	10	1	JCK-3
ugUaucuuguucgacagcacu	1	1	LT-1
Cggaucuuguucgacagcacu	2	1	JCK-3
uggaucuuguucgacaUcacu	5	1	JCK-2
uggaucuuguucgCcagcacu	10	1	LT-3
uggGucuuguucgacagcacu	9	1	JCK-1
uggaucuuguucgacagcacA	79	1	JCK-2
uggaucuCguucgacagcacu	1	1	JCK-1
uggaucuuguucgacGgcacu	1	1	LT-1

```
                                                                                                                          Mature                                                                   Star
uguuuucaucaacaguuagguuaaaaagaaaaugagcuccucuuauuuugucaucaaguauaaaaugacggcgagcucuuacagaugaaagcagugcgguuccaucuuguucguuuacccucuggaucuuguucgacagcacucuccuauauuuugccauuuaucuuuaaaugcgaaaucuuuaaaugcaaaauggcgacgaugauaggagugcugucgacaugauccagaaggugcagucgaauaagau
..........................................................................................................................uggaucuuguucgacagcacC...........................................................................................................   2      1    JT-2
..........................................................................................................................uggaucuuguucgacaAcacu...........................................................................................................   1      1    LT-1
..........................................................................................................................uggaucuuNuucgacagcacu...........................................................................................................   2      1    JCK-3
..........................................................................................................................uggaucuuguucgacCgcacu...........................................................................................................   3      1    LT-2
..........................................................................................................................uggaucuuguucgacagcaUu...........................................................................................................   18     1    LT-1
..........................................................................................................................uggaucuuguucgacagcaUu...........................................................................................................   17     1    LT-2
..........................................................................................................................uggaucuuguucgacagGacu...........................................................................................................   7      1    JCK-1
..........................................................................................................................uggaucuugCucgacagcacu...........................................................................................................   3      1    JCK-1
..........................................................................................................................uNgaucuuguucgacagcacu...........................................................................................................   29     1    JCK-3
..........................................................................................................................uggaucuuguucgacagcacu...........................................................................................................   277    0    JT-2
..........................................................................................................................uCgaucuuguucgacagcacu...........................................................................................................   3      1    JCK-2
..........................................................................................................................uggaucuuguucAacagcacu...........................................................................................................   7      1    JCK-2
..........................................................................................................................uggaucuuguucgacagcaAu...........................................................................................................   12     1    JCK-1
..........................................................................................................................uggaucuuguucgacagcaUu...........................................................................................................   7      1    JT-1
..........................................................................................................................uggaucuuguucgaNagcacu...........................................................................................................   4      1    LCK-1
..........................................................................................................................uggaucuuguucgacagcacA...........................................................................................................   50     1    LT-2
..........................................................................................................................uggaucuuguucgacGgcacu...........................................................................................................   1      1    LCK-2
..........................................................................................................................uggaucuuguucgacCgcacu...........................................................................................................   3      1    LT-3
..........................................................................................................................Cggaucuuguucgacagcacu...........................................................................................................   2      1    LT-3
..........................................................................................................................uggCucuuguucgacagcacu...........................................................................................................   67     1    JCK-1
..........................................................................................................................uggaucuuguucgacGgcacu...........................................................................................................   1      1    LCK-3
..........................................................................................................................uggaucuuguuNgacagcacu...........................................................................................................   1      1    JCK-2
..........................................................................................................................uggaucuuguucgCcagcacu...........................................................................................................   2      1    JT-3
..........................................................................................................................uggNucuuguucgacagcacu...........................................................................................................   21     1    JCK-1
..........................................................................................................................uggCucuuguucgacagcacu...........................................................................................................   19     1    LT-3
..........................................................................................................................uggaucCuguucgacagcacu...........................................................................................................   2      1    LCK-3
..........................................................................................................................uggaucuuguucgaUagcacu...........................................................................................................   2      1    LT-3
..........................................................................................................................uggaucuuguucgacagcUcu...........................................................................................................   1      1    JCK-2
..........................................................................................................................Aggaucuuguucgacagcacu...........................................................................................................   1      1    JCK-2
..........................................................................................................................uggaucuuguucgacagcaUu...........................................................................................................   13     1    JCK-1
..........................................................................................................................uggaucuuguucgacagcaUu...........................................................................................................   17     1    LT-3
..........................................................................................................................Aggaucuuguucgacagcacu...........................................................................................................   4      1    LT-2
..........................................................................................................................uUgaucuuguucgacagcacu...........................................................................................................   2      1    LCK-3
..........................................................................................................................uggaucuuguucgacagcacu...........................................................................................................   1125   0    JT-1
..........................................................................................................................uggaucuuguucgacGgcacu...........................................................................................................   2      1    JCK-1
..........................................................................................................................uggaucuuguucgacagUacu...........................................................................................................   1      1    LT-1
..........................................................................................................................uggaucuuAuucgacagcacu...........................................................................................................   1      1    LT-2
..........................................................................................................................uggNucuuguucgacagcacu...........................................................................................................   1      1    LCK-1
..........................................................................................................................uggaucuuguucgaUagcacu...........................................................................................................   12     1    JCK-1
..........................................................................................................................uggaucuuguucgacCgcacu...........................................................................................................   1      1    LCK-2
..........................................................................................................................uggaucuuguucgNcagcacu...........................................................................................................   5      1    LCK-1
..........................................................................................................................uggaucuuguucgacagcGcu...........................................................................................................   2      1    JCK-3
..........................................................................................................................uggaucuuguucgCcagcacu...........................................................................................................   8      1    LT-1
..........................................................................................................................uggaCcuuguucgacagcacu...........................................................................................................   1      1    LCK-2
..........................................................................................................................uggaucuuguucgaAagcacu...........................................................................................................   9      1    JCK-3
..........................................................................................................................uggaucuuguucgaUagcacu...........................................................................................................   26     1    JCK-2
..........................................................................................................................uggaucuuguucgacagcCcu...........................................................................................................   2      1    LCK-3
..........................................................................................................................uggaucuuguucgacagcaAu...........................................................................................................   3      1    JCK-2
..........................................................................................................................uggauNuuguucgacagcacu...........................................................................................................   1      1    LCK-1
..........................................................................................................................uggaCcuuguucgacagcacu...........................................................................................................   1      1    JCK-1
..........................................................................................................................uggaucuuguucgacagcacG...........................................................................................................   6      1    LCK-3
..........................................................................................................................uggaucAuguucgacagcacu...........................................................................................................   1      1    JCK-2
..........................................................................................................................uggaucuuguucCacagcacu...........................................................................................................   4      1    LCK-3
..........................................................................................................................uggaucuuguucgacagcCcu...........................................................................................................   3      1    LT-1
..........................................................................................................................uggaucuuguucgacaAcacu...........................................................................................................   8      1    JCK-1
..........................................................................................................................uUgaucuuguucgacagcacu...........................................................................................................   6      1    JCK-3
..........................................................................................................................uggaucuuguucgacagUacu...........................................................................................................   16     1    JCK-1
..........................................................................................................................uggaucuuguuNgacagcacu...........................................................................................................   1      1    JT-1
..........................................................................................................................uggaucuuguucgCcagcacu...........................................................................................................   16     1    LT-2
..........................................................................................................................uggauUuuguucgacagcacu...........................................................................................................   4      1    JCK-3
..........................................................................................................................uggaucuuguucgacagcGcu...........................................................................................................   1      1    JCK-1
..........................................................................................................................uggCucuuguucgacagcacu...........................................................................................................   10     1    LT-1
..........................................................................................................................uggaucuuguucUacagcacu...........................................................................................................   1      1    JCK-1
..........................................................................................................................uggaucuuguucgacCgcacu...........................................................................................................   7      1    JCK-1
..........................................................................................................................uNgaucuuguucgacagcacu...........................................................................................................   2      1    LT-3
..........................................................................................................................uggaucuuguucgacagcaAu...........................................................................................................   2      1    JCK-3
..........................................................................................................................uggaucuuguucUacagcacu...........................................................................................................   6      1    JCK-3
..........................................................................................................................uggaucuuguucgaAagcacu...........................................................................................................   1      1    JCK-1
..........................................................................................................................uggaucuuguucAacagcacu...........................................................................................................   6      1    JCK-1
..........................................................................................................................uggaucuuguucgacCgcacu...........................................................................................................   13     1    JCK-2
```

Mature   Star

uguuuucaucaacaguuagguuaaaaagaaaaugagcuccucuuauuuugucaucaaguauaaaaugacggcgagcucuuacagaugaaagcagugcgguuccaucuuguucguuuacccucuggaucuuguucgacagcacucuccuauauuuugccauuuaucuuuaaaugcgaaaucuuuaaaugcaaaauggcgacgaugauaggagugcugucgacaugauccagaaggugcagucgaauaagau

uggNucuuguucgacagcacu	1	1	JCK-2
uggaucuuguucgacaAcacu	1	1	LCK-3
uggaucuuguucgacagcacC	3	1	LT-3
uggaucuuguucgacagUacu	19	1	JCK-3
uggaucuuguucgacagAacu	1	1	JCK-2
uggaucuuguucgacagcaUu	38	1	JCK-2
uggauNuuguucgacagcacu	1	1	JCK-1
uggaCcuuguucgacagcacu	5	1	JCK-2
uggauUuuguucgacagcacu	1	1	JCK-1
uCgaucuuguucgacagcacu	1	1	LT-2
uggaucuuguCcgacagcacu	1	1	JCK-3
uggaucuuguucgacagcaAu	2	1	LT-3
ugAaucuuguucgacagcacu	6	1	JCK-1
uggaucuuguucgacagcacC	22	1	JCK-2
uggCucuuguucgacagcacu	2	1	LCK-2
uggaucuuguucgacagcacA	51	1	JCK-1
uggaucuuguucgacagcacC	1	1	LT-1
uNgaucuuguucgacagcacu	37	1	JCK-2
uggaucuuguucgacagcacC	5	1	JCK-1
uggaucuuguucgacagcaUu	38	1	LCK-3
ugAaucuuguucgacagcacu	11	1	JCK-3
uggaucuuguucgacagcacG	2	1	LT-1
uggaucuuguucUacagcacu	4	1	JCK-2
uggaucuuguCcgacagcacu	1	1	LT-3
uggaucuuAuucgacagcacu	1	1	LT-3
uggaucuuguucgacagcCcu	24	1	JCK-2
uggaucuuguucgacagNacu	3	1	JCK-3
uggaucuuguucgCcagcacu	2	1	JT-1
Cggaucuuguucgacagcacu	1	1	LCK-3
uggaucuuguucgGcagcacu	2	1	JCK-1
uggaucuuguucgacagcCcu	17	1	JCK-3
uggaucuuguucgacagcacu	5544	0	LCK-3
uggaucuuguucgacagcacG	12	1	JCK-1
uggaucuuguucgacagcUcu	2	1	LT-1
uggaucuuguucgacagcGcu	7	1	JCK-2
Cggaucuuguucgacagcacu	8	1	JCK-2
uggaucuuguucgacagcaGu	1	1	JCK-1
uggaucuuguucgacagcaAu	3	1	LT-2
uggaucuuguucgacagcaUu	17	1	JCK-3
uggaucuuAuucgacagcacu	10	1	JCK-2
uggaucuuguucgacagcacC	8	1	LT-2
uggaucuuguuAgacagcacu	1	1	LCK-3
uggaCcuuguucgacagcacu	4	1	JCK-3
uggaucuuguGcgacagcacu	3	1	JCK-1
ugCaucuuguucgacagcacu	1	1	JCK-1
uAgaucuuguucgacagcacu	10	1	JCK-2
uggaucuuAuucgacagcacu	1	1	JCK-3
uggaucuuguucgGcagcacu	2	1	JCK-3
uggaucuuguucgacagcacu	2933	0	LT-1
uggNucuuguucgacagcacu	4	1	JCK-3
uAgaucuuguucgacagcacu	3	1	LT-3
uggaucuuguucgacagcacG	12	1	JCK-3
uggaucuuguucgacGgcacu	5	1	JCK-2
uAgaucuuguucgacagcacu	3	1	LT-2
uggaucuuguucgacagcacu	20810	0	JCK-1
uUgaucuuguucgacagcacu	1	1	LT-2
uggaucCuguucgacagcacu	5	1	JCK-3
uCgaucuuguucgacagcacu	1	1	JCK-3
ugNaucuuguucgacagcacu	9	1	LT-2
uggaucCuguucgacagcacu	1	1	JT-1
uggGucuuguucgacagcacu	4	1	JCK-3
uAgaucuuguucgacagcacu	2	1	LCK-2
uggaucuuguucgacagcUcu	1	1	JCK-3
uggaucuuguucgacaAcacu	9	1	JCK-2
ugNaucuuguucgacagcacu	1	1	LT-1
uNgaucuuguucgacagcacu	31	1	JCK-1
uggCucuuguucgacagcacu	18	1	LCK-3
uggaucuuguuGgacagcacu	1	1	JCK-2
ugUaucuuguucgacagcacu	1	1	LCK-3
uggaucuuguucgacaUcacu	2	1	JCK-1

```
                                                                                                                          Mature                                                                                   Star
uguuuucaucaacaguuagguuaaaaagaaaaugagcuccucuuauuuugucaucaaguauaaaaugacggcgagcucuuacagaugaaagcagugcgguuccaucuuguucguuuacccucuggaucuuguucgacagcacucuccuauauuuugccauuuaucuuuaaaugcgaaaucuuuaaaugcaaaauggcgacgaugauaggagugcugucgacaugauccagaaggugcagucgaauaagau
..........................................................................................................................uggCucuuguucgacagcacu...........................................................................................................  75      1    JCK-3
..........................................................................................................................Gggaucuuguucgacagcacu...........................................................................................................  1       1    JCK-3
..........................................................................................................................uggaucuuguucgacaAcacu...........................................................................................................  2       1    JCK-3
..........................................................................................................................uggaucuuguucNacagcacu...........................................................................................................  1       1    LCK-1
..........................................................................................................................ugUaucuuguucgacagcacu...........................................................................................................  3       1    JCK-2
..........................................................................................................................uggaucuugCucgacagcacu...........................................................................................................  6       1    LCK-3
..........................................................................................................................uggaucuuguucgaAagcacu...........................................................................................................  5       1    JCK-2
..........................................................................................................................Cggaucuuguucgacagcacu...........................................................................................................  2       1    JCK-1
..........................................................................................................................uggGucuuguucgacagcacu...........................................................................................................  1       1    LCK-3
..........................................................................................................................ugUaucuuguucgacagcacu...........................................................................................................  2       1    JCK-1
..........................................................................................................................uggaucuuguucgacagcNcu...........................................................................................................  10      1    JCK-1
..........................................................................................................................uggaucuuguCcgacagcacu...........................................................................................................  2       1    JCK-2
..........................................................................................................................uggaucuuguucgacagcCcu...........................................................................................................  1       1    LCK-1
..........................................................................................................................uAgaucuuguucgacagcacu...........................................................................................................  9       1    JCK-1
..........................................................................................................................uUgaucuuguucgacagcacu...........................................................................................................  1       1    JCK-1
..........................................................................................................................uggCucuuguucgacagcacu...........................................................................................................  104     1    JCK-2
..........................................................................................................................Cggaucuuguucgacagcacu...........................................................................................................  2       1    LT-2
..........................................................................................................................uAgaucuuguucgacagcacu...........................................................................................................  7       1    LCK-3
..........................................................................................................................uggaucuuguucgaUagcacu...........................................................................................................  2       1    LT-1
..........................................................................................................................uggaucuuguucgacaUcacu...........................................................................................................  4       1    JCK-3
..........................................................................................................................uggaucuuguucgacagcacu...........................................................................................................  4196    0    LT-2
..........................................................................................................................uggaucuuUuucgacagcacu...........................................................................................................  3       1    JCK-3
..........................................................................................................................uggaucuuguucgacagcacu...........................................................................................................  35956   0    JCK-2
..........................................................................................................................uggaucCuguucgacagcacu...........................................................................................................  1       1    JT-3
..........................................................................................................................uggaucuuguucgGcagcacu...........................................................................................................  2       1    LCK-1
..........................................................................................................................uggGucuuguucgacagcacu...........................................................................................................  3       1    JCK-2
..........................................................................................................................uggaucuuguucgacaCcacu...........................................................................................................  1       1    JCK-1
..........................................................................................................................uggaucuuguucgacagcacA...........................................................................................................  16      1    LT-1
..........................................................................................................................uggaucuuguuGgacagcacu...........................................................................................................  1       1    JCK-3
..........................................................................................................................ugUaucuuguucgacagcacu...........................................................................................................  7       1    JCK-3
..........................................................................................................................uggaucGuguucgacagcacu...........................................................................................................  1       1    JCK-3
..........................................................................................................................uggaucuCguucgacagcacu...........................................................................................................  2       1    JCK-3
..........................................................................................................................uggaCcuuguucgacagcacu...........................................................................................................  1       1    LCK-3
..........................................................................................................................uggaucuuguuGgacagcacu...........................................................................................................  10      1    JCK-1
..........................................................................................................................uggaucCuguucgacagcacu...........................................................................................................  7       1    JCK-2
..........................................................................................................................uggaucuuguucAacagcacu...........................................................................................................  1       1    LCK-1
..........................................................................................................................uUgaucuuguucgacagcacu...........................................................................................................  3       1    JCK-2
..........................................................................................................................uggaucuuguucgCcagcacu...........................................................................................................  48      1    JCK-3
..........................................................................................................................uggaucuuguucgacagcacu...........................................................................................................  1264    0    LCK-1
..........................................................................................................................uggaucuuguucgacagcacA...........................................................................................................  24      1    LT-3
..........................................................................................................................uggaucuuCuucgacagcacu...........................................................................................................  1       1    JCK-3
..........................................................................................................................uggaucuuguucgacagcacA...........................................................................................................  59      1    JCK-3
..........................................................................................................................uggaucuuguucgacagcaAu...........................................................................................................  3       1    LT-1
..........................................................................................................................uggaucuuguucgacagUacu...........................................................................................................  23      1    JCK-2
..........................................................................................................................uggaucuuguucgaGagcacu...........................................................................................................  1       1    JCK-3
..........................................................................................................................uggaucCuguucgacagcacu...........................................................................................................  2       1    JCK-1
..........................................................................................................................uggaucuuguucgaUagcacu...........................................................................................................  11      1    JCK-3
..........................................................................................................................uggaucuuguucgacagcacA...........................................................................................................  37      1    JT-1
..........................................................................................................................uggaucuuguuGgacagcacu...........................................................................................................  1       1    LT-3
..........................................................................................................................uggaucuuguucgacagcacu...........................................................................................................  423     0    JT-3
..........................................................................................................................uAgaucuuguucgacagcacu...........................................................................................................  7       1    JCK-3
..........................................................................................................................ugAaucuuguucgacagcacu...........................................................................................................  2       1    LT-1
..........................................................................................................................uggCucuuguucgacagcacu...........................................................................................................  4       1    JT-1
..........................................................................................................................uggaucuuguucgacaUcacu...........................................................................................................  1       1    LT-2
..........................................................................................................................uggaucuugCucgacagcacu...........................................................................................................  9       1    JCK-2
..........................................................................................................................uggaucuuguucgacGgcacu...........................................................................................................  10      1    JCK-3
..........................................................................................................................uggaucuuguuNgacagcacu...........................................................................................................  121     1    JCK-1
..........................................................................................................................uggaucuuguucgacagcCcu...........................................................................................................  7       1    LT-2
..........................................................................................................................ugAaucuuguucgacagcacu...........................................................................................................  2       1    LT-2
..........................................................................................................................ugAaucuuguucgacagcacu...........................................................................................................  1       1    LT-3
..........................................................................................................................uggaucuuguucgacagcGcu...........................................................................................................  1       1    LT-3
..........................................................................................................................ugAaucuuguucgacagcacu...........................................................................................................  8       1    JCK-2
..........................................................................................................................uggaucuuguucgacagcacu...........................................................................................................  1314    0    LCK-2
..........................................................................................................................uggaucuuguucgacagcacu...........................................................................................................  26265   0    JCK-3
..........................................................................................................................uggCucuuguucgacagcacu...........................................................................................................  1       1    LCK-1
..........................................................................................................................uAgaucuuguucgacagcacu...........................................................................................................  1       1    JT-1
..........................................................................................................................uggaucCuguucgacagcacu...........................................................................................................  2       1    LT-2
..........................................................................................................................uggaucuuguucgacagcaAu...........................................................................................................  1       1    LCK-2
..........................................................................................................................uggaucuuguucAacagcacu...........................................................................................................  1       1    JCK-3
..........................................................................................................................uggaucuugCucgacagcacu...........................................................................................................  3       1    JCK-3
```

Mature / **Star**

```
uguuuucaucaacaguuagguuaaaaagaaaaugagcuccucuuauuuugucaucaaguauaaaaugacggcgagcucuuacagaugaaagcagugcgguuccaucuuguucguuuacccucuggaucuuguucgacagcacucuccuauauuuugccauuuaucuuuaaaugcgaaaucuuuaaaugcaaaauggcgacgaugauaggagugcugucgacaugauccagaaggugcagucgaauaagau
```

uggaucuCguucgacagcacu	1	1	LT-1
uggauUuuguucgacagcacu	4	1	JCK-2
ugAaucuuguucgacagcacu	2	1	LCK-3
uggaucuuguucgCcagcacu	2	1	LCK-2
uggaucuuguucgacagcCcu	2	1	LT-3
uggaucuuguucgacagcaAu	1	1	LCK-3
uggaucuuguucgacagcacu	4453	0	LT-3
uggaucuuguucgacagcacG	15	1	JCK-2
uggaucuuguucgCcagcacu	6	1	LCK-3
uggaucuuguuAgacagcacu	1	1	JCK-2
uggaucuuAuucgacagcacu	3	1	LCK-3
uggaucuCguucgacagcacu	7	1	JCK-2
uggaucuuguucgacagcCcu	11	1	JCK-1
uggaucuuUuucgacagcacu	3	1	JCK-2
uggaucuuguucgacagcacN	6	1	JCK-1
uggaucuuguucgacagcacC	2	1	LCK-3
uggaucuuguucgacagcacC	4	1	JT-3
uggaucuuguucAacagcacu	1	1	LCK-2
uggaucuuguCcgacagcacu	2	1	JCK-1
uggaucuuguucgCcagcacu	47	1	JCK-1
uggCucuuguucgacagcacu	55	1	LT-2
uggaucuuAuucgacagcacu	2	1	JCK-1
uUgaucuuguucgacagcacuc	1	1	LT-1
Cggaucuuguucgacagcacuc	3	1	JCK-2
uggaucuuguucgacagcacuc	58	0	JT-2
uggaucuuguucgacGgcacuc	4	1	JCK-3
uggaucuuguucgacaAcacuc	1	1	LT-3
uggaucuuAuucgacagcacuc	1	1	LT-3
uggaucuugCucgacagcacuc	5	1	JCK-2
uggaCcuuguucgacagcacuc	1	1	LT-1
uggauUuuguucgacagcacuc	2	1	JCK-3
uAgaucuuguucgacagcacuc	1	1	LCK-3
uggaucuuguucgacagcacuU	1	1	LCK-1
uggaucuuguucgacaAcacuc	1	1	LT-1
uggaucuuguucgacagUacuc	4	1	JCK-1
ugAaucuuguucgacagcacuc	2	1	JCK-1
uggaucuuguucgacagcaUuc	14	1	JCK-2
uNgaucuuguucgacagcacuc	15	1	JCK-2
uggaucuuguucgacagcacuU	25	1	LT-2
uggaucuuguucgaAagcacuc	5	1	JCK-2
uNgaucuuguucgacagcacuc	1	1	LT-3
uggaucuuguucgacagcCcuc	1	1	JCK-3
uggaucuuguucgacagcacuG	3	1	LT-1
uggaucuuguucgGcagcacuc	1	1	LCK-1
uggaucuuguucgacaAcacuc	1	1	JCK-1
uggaucCuguucgacagcacuc	2	1	LCK-3
uggaucuuguucgaUagcacuc	10	1	JCK-1
uggCucuuguucgacagcacuc	3	1	LT-1
uggaucuuguucgacagUacuc	5	1	JCK-2
uggaucuuguucgacagcacuG	2	1	JCK-3
Cggaucuuguucgacagcacuc	2	1	JCK-1
uggaucuuguucgacagcCcuc	1	1	LT-2
uggaucuuguucgacagUacuc	3	1	JCK-3
uggaucuuguucgUcagcacuc	1	1	JCK-3
uggCucuuguucgacagcacuc	7	1	LT-3
uggaucuuguucgacGgcacuc	2	1	LT-2
uggaucuuguucgacaCcacuc	1	1	LT-1
uggaucuuguucgacagcacuA	47	1	JCK-1
uggaucuuguucgacagcacAc	1	1	JCK-2
uggNucuuguucgacagcacuc	1	1	JCK-3
uggaucuuguucgacagcacuA	9	1	JT-1
uggaucuuguuGgacagcacuc	3	1	JCK-1
uggaucuuguucgUcagcacuc	3	1	JCK-2
uNgaucuuguucgacagcacuc	9	1	JCK-3
uggaucuuguucgacagcacuc	2355	0	LT-3
uggaucuuguucgacCgcacuc	1	1	LCK-2
uggaucuuguucgacagcacuN	4	1	JCK-1
uggaucuuguucgaUagcacuc	2	1	LT-1
uggaucuuguucAacagcacuc	1	1	LT-2
uggCucuuguucgacagcacuc	24	1	JCK-3

```
                                                                                                                          Mature                                                                                            Star
uguuuucaucaacaguuagguuaaaaagaaaaugagcuccucuuauuuugucaucaaguauaaaaugacggcgagcucuuacagaugaaagcagugcgguuccaucuuguucguuuacccucuggaucuuguucgacagcacucuccuauauuuugccauuuaucuuuaaaugcgaaaucuuuaaaugcaaaauggcgacgauaggagugcugucgacaugauccagaaggugcagucgaauaagau
..........................................................................................................................uggaucuuguucgCcagcacuc.......................................................................................................   17     1   JCK-1
..........................................................................................................................uggaucuuguucgacagcaAuc.......................................................................................................   1      1   JCK-1
..........................................................................................................................uggaucuuguucgacagcCcuc.......................................................................................................   3      1   JCK-1
..........................................................................................................................uggaucuuguucUacagcacuc.......................................................................................................   2      1   JCK-3
..........................................................................................................................ugAaucuuguucgacagcacuc.......................................................................................................   4      1   JCK-2
..........................................................................................................................uggaucuuguucgacaAcacuc.......................................................................................................   4      1   JCK-2
..........................................................................................................................uggaucuuguucgacagcacuG.......................................................................................................   9      1   JCK-1
..........................................................................................................................uggGucuuguucgacagcacuc.......................................................................................................   1      1   LT-3
..........................................................................................................................uggaucuuguucgacagcacuc.......................................................................................................   69     0   JT-3
..........................................................................................................................uggCucuuguucgacagcacuc.......................................................................................................   8      1   LT-2
..........................................................................................................................uUgaucuuguucgacagcacuc.......................................................................................................   1      1   JCK-1
..........................................................................................................................uAgaucuuguucgacagcacuc.......................................................................................................   3      1   JCK-1
..........................................................................................................................uggaucuuguucgacagcacuU.......................................................................................................   31     1   LCK-3
..........................................................................................................................uggaucuuguGcgacagcacuc.......................................................................................................   5      1   JCK-1
..........................................................................................................................uggaucuuguucgacagcacuU.......................................................................................................   81     1   JCK-3
..........................................................................................................................uggaucuuguucgCcagcacuc.......................................................................................................   29     1   JCK-2
..........................................................................................................................uggaucuuguucgacagcaNuc.......................................................................................................   5      1   JCK-1
..........................................................................................................................uggauUuuguucgacagcacuc.......................................................................................................   3      1   JCK-1
..........................................................................................................................uggaucuuguucgacagcacGc.......................................................................................................   1      1   JCK-1
..........................................................................................................................uggaucuuguucgCcagcacuc.......................................................................................................   11     1   JCK-3
..........................................................................................................................ugAaucuuguucgacagcacuc.......................................................................................................   5      1   LT-3
..........................................................................................................................uggaucuuguucUacagcacuc.......................................................................................................   2      1   LT-1
..........................................................................................................................uggaucuuguucgacagcaUuc.......................................................................................................   1      1   LT-1
..........................................................................................................................uAgaucuuguucgacagcacuc.......................................................................................................   3      1   JCK-3
..........................................................................................................................uggaCcuuguucgacagcacuc.......................................................................................................   2      1   JCK-2
..........................................................................................................................uggaucuuguucgacGgcacuc.......................................................................................................   2      1   JCK-2
..........................................................................................................................uggaucuuguucUacagcacuc.......................................................................................................   1      1   JCK-2
..........................................................................................................................uggaucuuguucgaAagcacuc.......................................................................................................   3      1   JCK-3
..........................................................................................................................uAgaucuuguucgacagcacuc.......................................................................................................   1      1   LT-3
..........................................................................................................................uggaucuugCucgacagcacuc.......................................................................................................   2      1   LT-1
..........................................................................................................................uggaucCuguucgacagcacuc.......................................................................................................   1      1   LT-3
..........................................................................................................................uggaucuuguucgacagcacuc.......................................................................................................   8479   0   JCK-1
..........................................................................................................................uggaucuuguucgacagcacuc.......................................................................................................   74     0   LCK-1
..........................................................................................................................uggaucuuguucgacagcacuc.......................................................................................................   1033   0   LT-2
..........................................................................................................................uggaucuuguucgUcagcacuc.......................................................................................................   1      1   JCK-1
..........................................................................................................................uggGucuuguucgacagcacuc.......................................................................................................   4      1   JCK-1
..........................................................................................................................uggaucuuguucUacagcacuc.......................................................................................................   2      1   JCK-1
..........................................................................................................................uggaucuuguucgaUagcacuc.......................................................................................................   26     1   JCK-3
..........................................................................................................................uggaucuuguucgacagcacCc.......................................................................................................   1      1   LT-3
..........................................................................................................................uggaucuuguucgacagcacNc.......................................................................................................   4      1   JCK-1
..........................................................................................................................uggaucuuguucgacCgcacuc.......................................................................................................   1      1   LT-3
..........................................................................................................................uggaucuuAuucgacagcacuc.......................................................................................................   1      1   JCK-1
..........................................................................................................................uUgaucuuguucgacagcacuc.......................................................................................................   1      1   JCK-2
..........................................................................................................................uggaucuuguucgacagcacuA.......................................................................................................   14     1   LCK-1
..........................................................................................................................uAgaucuuguucgacagcacuc.......................................................................................................   2      1   LT-1
..........................................................................................................................uggaucuuguucgCcagcacuc.......................................................................................................   5      1   LT-3
..........................................................................................................................uggaucuuguucgacagcacuU.......................................................................................................   120    1   JCK-2
..........................................................................................................................uggaucuuguucgacCgcacuc.......................................................................................................   8      1   JCK-2
..........................................................................................................................uggaucuuguucgacagcacCc.......................................................................................................   2      1   JCK-3
..........................................................................................................................uggaucuuguucgacagcNcuc.......................................................................................................   3      1   JCK-1
..........................................................................................................................uggaucuuguucCacagcacuc.......................................................................................................   1      1   JCK-3
..........................................................................................................................uggaucuuguucAacagcacuc.......................................................................................................   1      1   JCK-2
..........................................................................................................................uggNucuuguucgacagcacuc.......................................................................................................   8      1   JCK-1
..........................................................................................................................uggaucuuguucgacagAacuc.......................................................................................................   1      1   JCK-1
..........................................................................................................................uggaucuuguucgacCgcacuc.......................................................................................................   3      1   JCK-1
..........................................................................................................................uggaucuuguucgacagcaUuc.......................................................................................................   10     1   JCK-3
..........................................................................................................................uggaucuuguucgacagGacuc.......................................................................................................   6      1   JCK-1
..........................................................................................................................uggaucuuguucgacagcacuA.......................................................................................................   12     1   LT-2
..........................................................................................................................uggaucuuguucgacagcacuA.......................................................................................................   36     1   LCK-3
..........................................................................................................................uggaucuuguucgacagcGcuc.......................................................................................................   1      1   JCK-3
..........................................................................................................................uggaucuuguucgacaUcacuc.......................................................................................................   1      1   JCK-3
..........................................................................................................................uAgaucuuguucgacagcacuc.......................................................................................................   4      1   JCK-2
..........................................................................................................................uggaucuuguucCacagcacuc.......................................................................................................   2      1   JCK-1
..........................................................................................................................uggaucuuUuucgacagcacuc.......................................................................................................   3      1   JCK-3
..........................................................................................................................uggaucuuguucgacagcacuc.......................................................................................................   188    0   JT-1
..........................................................................................................................uggaucuuUuucgacagcacuc.......................................................................................................   1      1   JCK-1
..........................................................................................................................Cggaucuuguucgacagcacuc.......................................................................................................   1      1   LT-2
..........................................................................................................................uggaucuuguucgacGgcacuc.......................................................................................................   1      1   JCK-1
..........................................................................................................................uggaucuuguucgacagcacuc.......................................................................................................   955    0   LCK-3
..........................................................................................................................uggaucuuguucgacagcacAc.......................................................................................................   1      1   LT-1
```

Mature Star

```
uguuuucaucaacaguuagguuaaaaagaaaaugagcuccucuuauuuugucaucaaguauaaaaugacggcgagcucuuacagaugaaagcagugcgguuccaucuuguucguuuacccucuggaucuuguucgacagcacucuccuauauuuugccauuuaucuuuaaaugcgaaaucuuuaaaugcaaaauggcgacgaugauaggagugcugucgacaugauccagaaggugcagucgaauaagau
..........................................................................................................................uggauUuuguucgacagcacuc..........................................................................................................   4      1   JCK-2
..........................................................................................................................uggaucuuguucgacagcacuA..........................................................................................................   19     1   LT-1
..........................................................................................................................uggaucuuguucgacagcacuU..........................................................................................................   27     1   LT-1
..........................................................................................................................ugAaucuuguucgacagcacuc..........................................................................................................   5      1   JCK-3
..........................................................................................................................Cggaucuuguucgacagcacuc..........................................................................................................   3      1   JCK-3
..........................................................................................................................uggaucuuUuucgacagcacuc..........................................................................................................   3      1   JCK-2
..........................................................................................................................uggaucuuguucgaUagcacuc..........................................................................................................   42     1   JCK-2
..........................................................................................................................uggaucuuguucCacagcacuc..........................................................................................................   5      1   JCK-2
..........................................................................................................................uggaucuuguucgacagcGcuc..........................................................................................................   2      1   JCK-1
..........................................................................................................................uggaCcuuguucgacagcacuc..........................................................................................................   3      1   JCK-3
..........................................................................................................................uggaucuuguucgacCgcacuc..........................................................................................................   5      1   JCK-3
..........................................................................................................................uggaucuuguucAacagcacuc..........................................................................................................   1      1   LT-1
..........................................................................................................................uggaucuuguucAacagcacuc..........................................................................................................   2      1   JCK-3
..........................................................................................................................uggaucuuguucgacagcacuc..........................................................................................................   14605  0   JCK-2
..........................................................................................................................uggaucuuguuNgacagcacuc..........................................................................................................   29     1   JCK-1
..........................................................................................................................uggaucuuguucgacagcacuA..........................................................................................................   67     1   JCK-3
..........................................................................................................................uggaucuuguucgacagcacuA..........................................................................................................   61     1   JCK-2
..........................................................................................................................uggaucuCguucgacagcacuc..........................................................................................................   1      1   LT-1
..........................................................................................................................uAgaucuuguucgacagcacuc..........................................................................................................   2      1   LT-2
..........................................................................................................................uggaucuuAuucgacagcacuc..........................................................................................................   1      1   JCK-3
..........................................................................................................................uggaucuuguucgacagGacuc..........................................................................................................   2      1   JCK-2
..........................................................................................................................uggaucuuguucgacagcCcuc..........................................................................................................   2      1   LT-1
..........................................................................................................................uggaCcuuguucgacagcacuc..........................................................................................................   1      1   JCK-1
..........................................................................................................................uggaucuuAuucgacagcacuc..........................................................................................................   2      1   JCK-2
..........................................................................................................................uggaucuuguucgacagcCcuc..........................................................................................................   9      1   JCK-2
..........................................................................................................................uggaucuuguucgacagcacuA..........................................................................................................   24     1   LT-3
..........................................................................................................................uggaucuuguucgacagcacuG..........................................................................................................   12     1   JCK-2
..........................................................................................................................uUgaucuuguucgacagcacuc..........................................................................................................   2      1   JCK-3
..........................................................................................................................uggaucuCguucgacagcacuc..........................................................................................................   1      1   LCK-3
..........................................................................................................................uggCucuuguucgacagcacuc..........................................................................................................   2      1   JT-1
..........................................................................................................................uggaucuCguucgacagcacuc..........................................................................................................   1      1   JCK-3
..........................................................................................................................uggaucuugCucgacagcacuc..........................................................................................................   5      1   JCK-1
..........................................................................................................................uggaucCuguucgacagcacuc..........................................................................................................   1      1   JCK-2
..........................................................................................................................uggaucuugCucgacagcacuc..........................................................................................................   4      1   JCK-3
..........................................................................................................................uggGucuuguucgacagcacuc..........................................................................................................   2      1   JCK-2
..........................................................................................................................uggaucuuNuucgacagcacuc..........................................................................................................   1      1   JCK-3
..........................................................................................................................uUgaucuuguucgacagcacuc..........................................................................................................   1      1   LCK-3
..........................................................................................................................uNgaucuuguucgacagcacuc..........................................................................................................   11     1   JCK-1
..........................................................................................................................uggaucuuguucgacaUcacuc..........................................................................................................   4      1   JCK-2
..........................................................................................................................uggaucuuguucgacagcacuU..........................................................................................................   63     1   JCK-1
..........................................................................................................................uggaucuuguucgacagNacuc..........................................................................................................   1      1   JCK-3
..........................................................................................................................uggaucuuguucgacagcacuc..........................................................................................................   10812  0   JCK-3
..........................................................................................................................uggCucuuguucgacagcacuc..........................................................................................................   1      1   LCK-3
..........................................................................................................................uggaucuuguucgacagcCcuc..........................................................................................................   1      1   LT-3
..........................................................................................................................uggaucCuguucgacagcacuc..........................................................................................................   3      1   JCK-3
..........................................................................................................................uggaucuCguucgacagcacuc..........................................................................................................   1      1   JCK-1
..........................................................................................................................uggaucuuguucgCcagcacuc..........................................................................................................   2      1   LT-1
..........................................................................................................................uggaucuuguucgacagcacCc..........................................................................................................   2      1   JCK-2
..........................................................................................................................uggaucuuguucgaUagcacuc..........................................................................................................   1      1   LT-3
..........................................................................................................................uggCucuuguucgacagcacuc..........................................................................................................   19     1   JCK-1
..........................................................................................................................uggaucuuguucgacagcacuc..........................................................................................................   84     0   LCK-2
..........................................................................................................................uNgaucuuguucgacagcacuc..........................................................................................................   1      1   LCK-3
..........................................................................................................................uggaucuuguucgacagcacuA..........................................................................................................   11     1   LCK-2
..........................................................................................................................ugNaucuuguucgacagcacuc..........................................................................................................   2      1   LT-2
..........................................................................................................................uggaucuuguucgCcagcacuc..........................................................................................................   1      1   LT-2
..........................................................................................................................uggaucuuguucgacagcaUuc..........................................................................................................   7      1   JCK-1
..........................................................................................................................uggCucuuguucgacagcacuc..........................................................................................................   43     1   JCK-2
..........................................................................................................................ugAaucuuguucgacagcacuc..........................................................................................................   1      1   LT-1
..........................................................................................................................uggaucuuguucgacagcacuG..........................................................................................................   1      1   LT-3
..........................................................................................................................uggaucuuguucAacagcacuc..........................................................................................................   1      1   JCK-1
..........................................................................................................................uggaucuuguCcgacagcacuc..........................................................................................................   1      1   JCK-3
..........................................................................................................................uggaucuuguucgacaUcacuc..........................................................................................................   1      1   JCK-1
..........................................................................................................................uggaucuuguucgacagcacuc..........................................................................................................   2023   0   LT-1
..........................................................................................................................uggaucuuguucgacagcacuU..........................................................................................................   35     1   LT-3
..........................................................................................................................uggaucuuguucgacGgcacuc..........................................................................................................   1      1   LT-1
..........................................................................................................................uggaucuuguucgacagcacuAu.........................................................................................................   1      1   LT-3
..........................................................................................................................uNgaucuuguucgacagcacucu.........................................................................................................   1      1   JCK-2
..........................................................................................................................uggaucuuguucgacagcacucu.........................................................................................................   860    0   JCK-1
..........................................................................................................................uggaucuuguucgacagcacuUu.........................................................................................................   8      1   JT-2
..........................................................................................................................uggaucuuguucgacagcacucu.........................................................................................................   15     0   LT-2
```

```
                                                                                                                          Mature                                                                                    Star
uguuuucaucaacaguuagguuaaaaagaaaaugagcuccucuuauuuugucaucaaguauaaaaugacggcgagcucuuacagaugaaagcagugcgguuccaucuuguucguuuacccucuggaucuuguucgacagcacucuccuauauuuugccauuuaucuuuaaaugcgaaaucuuuaaaugcaaaauggcgacgaugauaggagugcugucgacaugauccagaaggugcagucgaauaagau
..........................................................................................................................uggaucuuguucgacagcacucG.........................................................................................................   1      1   LT-3
..........................................................................................................................uggaucuuguucgacagcacuAu.........................................................................................................   3      1   LT-1
..........................................................................................................................uggaucuuguucgacagcacucu.........................................................................................................   1104   0   JCK-3
..........................................................................................................................uggaucuuguucgacagcacuUu.........................................................................................................   17     1   LCK-2
..........................................................................................................................uggaucuuguucgacagcacuAu.........................................................................................................   1      1   JCK-1
..........................................................................................................................uggaucuuguucgacagcacuUu.........................................................................................................   5      1   JT-3
..........................................................................................................................uAgaucuuguucgacagcacucu.........................................................................................................   1      1   JCK-3
..........................................................................................................................uggaucuuguucgacagcacuUu.........................................................................................................   21     1   LT-1
..........................................................................................................................uggaucuuguucgacagcacucA.........................................................................................................   8      1   LT-2
..........................................................................................................................uggaucuuguucgaUagcacucu.........................................................................................................   2      1   JCK-2
..........................................................................................................................ugAaucuuguucgacagcacucu.........................................................................................................   1      1   JCK-1
..........................................................................................................................uggaucCuguucgacagcacucu.........................................................................................................   1      1   JCK-2
..........................................................................................................................uNgaucuuguucgacagcacucu.........................................................................................................   2      1   JCK-1
..........................................................................................................................uggaucuuguucgacagcacucN.........................................................................................................   1      1   JCK-3
..........................................................................................................................uggaucuuguucgacagcacucA.........................................................................................................   14     1   LT-1
..........................................................................................................................uggaucuuguucgacagcacuAu.........................................................................................................   2      1   JCK-2
..........................................................................................................................uggaucuuguucgacCgcacucu.........................................................................................................   1      1   JCK-3
..........................................................................................................................uNgaucuuguucgacagcacucu.........................................................................................................   1      1   JCK-3
..........................................................................................................................uggaucuuguucgacagcacuUu.........................................................................................................   50     1   LCK-3
..........................................................................................................................uggaucuuguucgacagcacCcu.........................................................................................................   1      1   JCK-2
..........................................................................................................................uggaucuuguucgacagUacucu.........................................................................................................   1      1   JCK-2
..........................................................................................................................uggCucuuguucgacagcacucu.........................................................................................................   3      1   JCK-2
..........................................................................................................................uggCucuuguucgacagcacucu.........................................................................................................   2      1   JCK-1
..........................................................................................................................uggaucuuguucgCcagcacucu.........................................................................................................   1      1   JCK-1
..........................................................................................................................uggaucuuguucAacagcacucu.........................................................................................................   1      1   JCK-3
..........................................................................................................................uggaucuuguucgacagcacucA.........................................................................................................   18     1   LCK-3
..........................................................................................................................uggaucuuCuucgacagcacucu.........................................................................................................   1      1   JCK-3
..........................................................................................................................uggaucuuguucgCcagcacucu.........................................................................................................   2      1   JCK-2
..........................................................................................................................uggaucuuguuNgacagcacucu.........................................................................................................   3      1   JCK-1
..........................................................................................................................uggaCcuuguucgacagcacucu.........................................................................................................   1      1   JCK-2
..........................................................................................................................uggaucuuguucgacagcacucA.........................................................................................................   59     1   JCK-1
..........................................................................................................................uggaucuuguucgaUagcacucu.........................................................................................................   2      1   JCK-3
..........................................................................................................................uggaucuuguucgacagcacucu.........................................................................................................   1493   0   JCK-2
..........................................................................................................................ugAaucuuguucgacagcacucu.........................................................................................................   1      1   JCK-3
..........................................................................................................................Cggaucuuguucgacagcacucu.........................................................................................................   1      1   JCK-1
..........................................................................................................................uggNucuuguucgacagcacucu.........................................................................................................   2      1   JCK-1
..........................................................................................................................uggaucuuguucgacagcaNucu.........................................................................................................   1      1   JCK-1
..........................................................................................................................uggaucuuguucgacagcacuUu.........................................................................................................   32     1   LCK-1
..........................................................................................................................uggaucuuguucgacagcacuGu.........................................................................................................   2      1   JCK-1
..........................................................................................................................uggaucuuguucgCcagcacucu.........................................................................................................   2      1   LT-3
..........................................................................................................................uggaucuuguucgacagcacucu.........................................................................................................   4      0   JT-1
..........................................................................................................................uggaucuuguucgacagcaUucu.........................................................................................................   1      1   JCK-1
..........................................................................................................................uggaucuuguucgacagcacucu.........................................................................................................   15     0   LCK-3
..........................................................................................................................uggaucuuguucgacagcacucA.........................................................................................................   52     1   JCK-3
..........................................................................................................................uggaucuuguucgacagcacuGu.........................................................................................................   1      1   LT-3
..........................................................................................................................uggaucuuguucgacagcacucG.........................................................................................................   5      1   LT-2
..........................................................................................................................uggaucuuguucgacagcacucA.........................................................................................................   64     1   JCK-2
..........................................................................................................................uggaucuuguucgacagcacuUu.........................................................................................................   52     1   JCK-3
..........................................................................................................................uggCucuuguucgacagcacucu.........................................................................................................   3      1   JCK-3
..........................................................................................................................uggCucuuguucgacagcacucu.........................................................................................................   2      1   LT-2
..........................................................................................................................uggaucuuguucgacagcacucG.........................................................................................................   1      1   JCK-3
..........................................................................................................................uggaucuuguucgacagcacuUu.........................................................................................................   20     1   LT-3
..........................................................................................................................uggaucuuNuucgacagcacucu.........................................................................................................   1      1   JCK-3
..........................................................................................................................uggaucuuguucgacagcacucu.........................................................................................................   27     0   LT-3
..........................................................................................................................uggaucuuguucgacagcacuAu.........................................................................................................   2      1   JCK-3
..........................................................................................................................uggaucuuguucgacagcacucu.........................................................................................................   19     0   JT-3
..........................................................................................................................uggaucuuguucgaAagcacucu.........................................................................................................   1      1   LT-1
..........................................................................................................................uggaucuuguucgacagcacucA.........................................................................................................   11     1   LT-3
..........................................................................................................................uggaucuuguucgacagcacuUu.........................................................................................................   52     1   JCK-1
..........................................................................................................................uggaucuuguucgaUagcacucu.........................................................................................................   5      1   JCK-1
..........................................................................................................................uggaucuuguucgacagcacuNu.........................................................................................................   2      1   JCK-1
..........................................................................................................................uggaucuuguucgacagcacucu.........................................................................................................   15     0   JT-2
..........................................................................................................................uggaucuuguucgCcagcacucu.........................................................................................................   3      1   JCK-3
..........................................................................................................................uggaucuuguucgacagcacuUu.........................................................................................................   70     1   JCK-2
..........................................................................................................................uggCucuuguucgacagcacucu.........................................................................................................   1      1   JT-1
..........................................................................................................................ugUaucuuguucgacagcacucu.........................................................................................................   1      1   JCK-2
..........................................................................................................................uggaucuuguucgacagcacucG.........................................................................................................   4      1   JCK-2
..........................................................................................................................uggaucuuguucgacagcacucu.........................................................................................................   14     0   LT-1
..........................................................................................................................uggaucuuguucgacagcacuUu.........................................................................................................   22     1   LT-2
..........................................................................................................................uggaucuuguucgacagcacucuU........................................................................................................   10     1   JCK-3
```

Mature (uggaucuuguucgacagcacu) — Star (gugcugucgacaugauccaga)

uguuuucaucaacaguuagguuaaaaagaaaaugagcuccucuuauuuugucaucaaguauaaaaugacggcgagcucuuacagaugaaagcagugcgguuccaucuuguucguuuacccucuggaucuuguucgacagcacucuccuauauuuugccauuuaucuuuaaaugcgaaaucuuuaaaugcaaaauggcgacgaugauaggagugcugucgacaugauccagaaggugcagucgaauaagau

................uggaucuuguucgacagcacucuU................	3	1	LT-2
................uggaucuuguucgacagcacucuc................	6	0	LT-2
................uggaucuuguucgacagcacucuA................	31	1	JCK-2
................uggaucuuguucgacagcacucuU................	12	1	JCK-1
................uggaucuuguuNgacagcacucuc................	1	1	JCK-1
................uggaucuuguucgacagcacuGuc................	3	1	JCK-2
................uggaucuuguucgacagcacucuU................	13	1	JCK-2
................uggaucuuguucgacagcacucuU................	1	1	LT-1
................uggaucuuguucgacagcacuUuc................	2	1	JCK-3
................uggaucuuguucgacagcacucuA................	1	1	LT-2
................uggaucuuguucgacagcacucuc................	7	0	LT-1
................uggaucuuguucgacagcacucuc................	10	0	JCK-3
................uggaucuuguucgacagcacucuA................	1	1	LCK-3
................uggaucuuguucgacagcacucuA................	3	1	LT-3
................uggaucuuguucgacagcacucuA................	17	1	JCK-1
................uggaucuuguucgacagcacucuG................	1	1	JCK-1
................uggaucuuguucgacagcacuUuc................	5	1	LCK-3
................uggaucuuguucgacagcacuAuc................	1	1	JCK-2
................uggaucuuguucgacagcacucuA................	22	1	JCK-3
................uggaucuuguucgacagcacucuc................	11	0	JCK-1
................uggaucuuguucgacagcacuUuc................	6	1	JCK-2
................uggaucuuguucgacagcacucuc................	5	0	LT-3
................uggaucuuguucgacagcacuUuc................	2	1	JCK-1
................uggaucuuguucgacagcacucuc................	10	0	JCK-2
................uggaucuuguucgacagcacucucc................	6	0	JCK-3
................uggaucuuguucgacagcacucucc................	17	0	LCK-1
................uggaucuuguucgacagcacucucc................	8	0	LT-3
................uggaucuuguucgacagcacucucc................	5	0	JCK-2
................uggaucuuguucgacagcacucucc................	15	0	LCK-2
................uggaucuuguucgacagcacucucc................	17	0	LT-1
................uggaucuuguucgacagcaNucucc................	1	1	JCK-1
................uggaucuuguucgacagcacucucc................	18	0	LT-2
................uggaucuuguucgacagcacucucA................	1	1	LT-3
................uggaucuuguucgacagcacucucc................	9	0	JCK-1
................uggaucuuguucgacagcacucuNc................	1	1	LT-2
................uggaucuuguucgacagcacucucc................	28	0	LCK-3
................uggaucuuguucgacagcacucuccu................	8	0	LT-3
................uggaucuuguucgacagcacucucGu................	1	1	JCK-3
................uggaucuuguucgacagcacucuccA................	1	1	LT-3
................uggaucuuguucgacagcacucucGu................	1	1	JCK-2
................uggaucuuguucgacagcacucuccu................	3	0	LT-1
................uggaucuuguucgacagcacucuccu................	24	0	LCK-3
................uggaucuuguucgacagcacucucAu................	1	1	LCK-3
................uggaucuuguucgacagcacucuccu................	1	0	JCK-1
................uggaucuuguucgacagcacucuccC................	1	1	LT-3
................uggaucuuguucgacagcacucuccu................	9	0	LT-2
................uggaucuuguucgacagcacucuccuU................	1	1	LT-2
................uggaucuuguucgacagcacucuccua................	2	0	LT-2
.................ggaucuuguucgacagca................	2	0	JCK-1
.................ggaucuuguucgacagca................	5	0	LT-2
.................ggaucuuguucgacagca................	10	0	JT-1
.................ggaucuuguucgacagcacu................	1	0	LT-2
.................ggaucuuguucgacagcacu................	2	0	JCK-1
.................ggaucuuguucgacagcacu................	5	0	JCK-3
.................Ugaucuuguucgacagcacu................	1	1	LT-1
.................ggaucuuguucgacagcacu................	1	0	LT-1
.................ggaucuuguucgacagcacu................	1	0	JCK-2
.................ggaucuuguucgacagcacuc................	1	0	LT-1
.................ggaucuuguucgacagcacuc................	2	0	JCK-3
.................ggaucuuguucgacagcacuc................	3	0	JCK-2
.................ggaucuuguucgacagcacucu................	2	0	JCK-3
.................ggaucuuguucgacagcacucu................	1	0	JCK-2
.................ggaucuuguucgacagcacucuccu................	2	0	LCK-3
.................ggaucuuguucgacagcacucuccuU................	1	1	LT-1
..................gaucuuguucgacagcacu................	2	0	JCK-3
..................gaucuuguucgacagcacu................	2	0	JCK-1
..................gaucuuguucgacagcacucu................	1	0	JCK-3
..................gaucuuguucgacagcacucuc................	3	0	JCK-3
..................gaucuuguucgacagcacucuc................	2	0	JCK-2
...................aucuuguucgacagcacu................	1	0	LT-3

Mature **Star**

uguuuucaucaacaguuagguuaaaaagaaaaugagcuccucuuauuuugucaucaaguauaaaaugacggcgagcucuuacagaugaaagcagugcgguuccaucuuguucguuuacccucuggaucuuguucgacagcacucuccuauauuuugccauuuaucuuuaaaugcgaaaucuuuaaaugcaaaauggcgacgaugauaggagugcugucgacaugauccagaaggugcagucgaauaagau

Mature: uggaucuuguucgacagcacu

Star: gugcugucgacaugauccaga

aucuuguucgacagcacuc	2	0	JCK-1
aucuuguucgacagcacuc	2	0	JCK-2
aucuuguucgacagcacuc	1	0	LT-3
aucuuguucgacagcacuc	3	0	JCK-3
aucuuguucgacagcacucu	2	0	JCK-2
aucuuguucgacagcacucu	1	0	LT-1
aucuuguucgacagcacuUu	2	1	JCK-3
aucuuguucgacagcacucu	1	0	LT-3
aucuuguucgacagcacucuc	1	0	JCK-2
aucuuguucgacagcacucuc	2	0	LT-3
aucuuguucgacagcacucuc	2	0	LT-1
aucuuguucgacagcacucuc	2	0	JCK-1
aucuuguucgaNagcacucuc	1	1	JCK-1
aucuuguucgacagcacucuc	1	0	JCK-3
aucuuguucgacagcacucucc	1	0	JCK-3
aucuuguucgacagcacucucc	1	0	JCK-2
aucuuguucgacagcacucucc	1	0	JCK-1
aucuuguucgacagcacucucc	3	0	LT-2
aucuuguucgacagcacucucUu	1	1	JCK-1
ucuuguucgacagcacuc	1	0	LT-1
ucuuguucgacagcacuc	2	0	LT-3
ucuuguucgacagcacucu	1	0	LT-3
ucuuguucgacagcacucuc	1	0	JCK-3
ucuuguucgacagcacucuccu	1	0	JCK-2
ucuuguucgacagcacucuccu	1	0	LT-1
ucuuguucgacagcacucuccu	1	0	LT-3
uggcgacgaugauaggagugcug	9	0	LCK-3
auaggagugcugucgacaugaucca	2	0	LT-2
auaggagugcugucgacaugauccagaa	1	0	LT-1
uaggagugcugucgacaugauccaga	6	0	LCK-3
uaggagugcugucgacaugauccaga	1	0	LT-1
aggagugcugucgacaug	2	0	LT-2
aggagugcugucgacaug	4	0	LCK-3
aggagugcugucgacauga	1	0	LT-1
aggagugcugucgacaugau	6	0	LT-3
aggagugcugucgacaugau	8	0	LT-2
aggagugcugucgacaugauU	1	1	LT-1
aggagugcugucgacaugauc	33	0	LT-3
aggagugcugucgCcaugauc	1	1	LT-2
aggagugcugucgCcaugauc	1	1	LT-1
aggagugcugucgacaugauc	19	0	LT-1
aggagugcugucgacaugauc	49	0	LCK-3
aggagugcugucgacaugCuc	1	1	LT-2
aggagugcugucgacaugauU	2	1	LT-2
aggagugcugucgacaugauU	1	1	LT-3
aggagugcugucgacaugauc	77	0	LT-2
Cggagugcugucgacaugauc	1	1	LT-3
aggagugcugucgacaugaucc	1	0	LT-1
aggagugcugucgacaugauccag	3	0	LCK-3
Gggagugcugucgacaugauccagaaggug	1	1	JCK-3
ggagugcugucgacaugauc	2	0	LT-3
ggagugcugucgacaugaucc	2	0	LT-2
ggagugcugucgacaugaucc	1	0	LT-3
ggagugcugucgacaugaucc	9	0	LCK-3
ggagugcugucgacaugaucca	1	0	LT-3
ggGgugcugucgacaugauccag	1	1	JCK-3
ggGgugcugucgacaugauccag	3	1	JCK-2
ggGgugcugucgacaugauccag	2	1	JCK-1
ggagugcugucgacaugauccaga	4	0	LT-1
ggagugcugucgacaugauccaga	2	0	JCK-2
ggagugcugucgacaugauccaga	2	0	LT-2
ggGgugcugucgacaugauccaga	15	1	JCK-2
ggGgugcugucgacaugauccaga	10	1	JCK-1
ggGgugcugucgacaugauccaga	20	1	JCK-3
ggagugcugucgacaugauccaga	8	0	LT-3
ggagugcugucgacaugauccaga	1	0	JCK-3
ggagugcugucgacaugauccagaa	2	0	LT-2
ggagugcugucgacaugauccagaC	1	1	LT-1
gagugcugucgacaugau	1	0	LT-3
gagugcugucgacaugauc	1	0	LT-2

Mature **Star**

uguuuucaucaacaguuagguuaaaaagaaaaugagcuccucuuauuuugucaucaaguauaaaaugacggcgagcucuuacagaugaaagcagugcgguuccaucuuguucguuuacccucuggaucuuguucgacagcacucuccuauauuuugccauuuaucuuuaaaugcgaaaucuuuaaaugcaaaauggcgacgaugauaggagugcugucgacaugauccagaaggugcagucgaauaagau

gagugcugucgacaugauc	5	0	LT-3
gagugcugucgacaugauc	1	0	LCK-3
gagugcugucgacaugauc	3	0	LT-1
Aagugcugucgacaugaucc	1	1	LCK-3
gagugcugucgacaAgaucc	8	1	JT-3
gagugcugucgacaugaucc	3	0	LT-2
gagugcugucgacaAgaucc	5	1	JT-2
gagugcugucgacaugaucc	12	0	LT-3
gagugcugucgacaugaucA	1	1	JCK-1
gagugcugucgacaugaucc	7	0	LT-1
gagugcugucgacaugaucU	1	1	LT-1
gagugcugucgacaAgaucc	9	1	JCK-2
gagugcugucgacaAgaucc	10	1	JCK-3
gagugcugucgacaAgaucc	2	1	JCK-1
gagugcugucgacaugaucc	8	0	LCK-3
gagugcugucgacaugaucc	2	0	JCK-3
gagugcugucgacaugaucca	4	0	LT-3
gCgugcugucgacaugaucca	1	1	LCK-1
gagugcugucgacaugaucca	27	0	LCK-1
gagugcugucgacaugaucca	14	0	LT-2
gagugcugucgacaugaucca	22	0	LCK-2
gagugcugucgacaAgaucca	15	1	JCK-2
gagugcugucgacaugaucca	3	0	LT-1
gagugcugucgacaugauccC	2	1	JCK-2
gagugcugucgacaAgaucca	8	1	JCK-3
gagugcugucgacaAgaucca	8	1	JCK-1
gagugcugucgacaugauccag	11	0	LT-1
gagugcugucgacaugauccag	2	0	LCK-3
gagugcugucgacaugauccag	3	0	LT-2
gagugcugucgacaugauccag	14	0	LT-3
gagugcugucgacaAgauccaga	72	1	JCK-2
gagugcugucgacaugauccaga	5	0	LT-1
gagugcugucgacaAgauccaga	35	1	JCK-1
gagugcugucgacaAgauccaga	44	1	JCK-3
gagugcugucgacaugauccaga	2	0	LT-2
agugcugucgacaugauc	1	0	JCK-3
Ggugcugucgacaugauc	1	1	JCK-3
agugcugucgacaugauc	3	0	JCK-1
Ggugcugucgacaugauc	2	1	JCK-2
agugcugucgacaugauc	5	0	LT-2
agugcugucgacaugauc	4	0	LT-1
agugcugucgacaugauc	4	0	LT-3
Ggugcugucgacaugaucc	1	1	JCK-1
Ggugcugucgacaugaucc	2	1	JCK-2
agugcugucgacaAgaucc	7	1	JCK-2
agugcugucgacaugaucc	3	0	LT-3
agugcugucgacaugaucc	4	0	LCK-3
agugcugucgacaugaucc	1	0	JCK-3
agugcugucgacaugaucc	1	0	LT-1
agugcugucgacaAgaucc	3	1	JCK-3
agugcugucgacaugaucca	2	0	LT-2
agugcugucgacaugaucca	2	0	LT-1
agugcugucgacaugaucca	10	0	LCK-3
agugcugucgacaAgaucca	9	1	JCK-2
agugcugucgacaAgaucca	2	1	JCK-1
agugcugucgacaAgaucca	4	1	JCK-3
agugcugucgacaugaucca	3	0	LT-3
agugcugucgacaAgauccag	2	1	JCK-2
agugcugucgacaugauccag	2	0	JCK-2
agugcugucgacaugauccag	6	0	LT-1
agugcugucgacaugauccag	8	0	LT-3
agugcugucgacaAgauccag	5	1	JCK-1
agugcugucgacaugauccag	1	0	JCK-1
agugcugucgacaAgauccag	4	1	JCK-3
agugcugucgacaugauccaC	4	1	LCK-3
agugcugucgacaugauccag	3	0	JCK-3
agugcugucgacaugauccag	8	0	LT-2
Cgugcugucgacaugauccaga	3	1	JCK-2
agugUugucgacaugauccaga	1	1	LT-2
agugcugucgacaugauccaga	14	0	JCK-2

Mature Star

uguuuucaucaacaguuagguuaaaaagaaaaugagcuccucuuauuuugucaucaaguauaaaaugacggcgagcucuuacagaugaaagcagugcgguuccaucuuguucguuuacccucuggaucuuguucgacagcacucuccuauauuuugccauuuaucuuuaaaugcgaaaucuuuaaaugcaaaauggcgacgaugauaggagugcugucgacaugauccagaaggugcagucgaauaagau

Cgugcugucgacaugauccaga	1	1	JCK-3
agugcugucgacaAgauccaga	8	1	JCK-2
agugcugucgacaugauccaga	34	0	LT-3
agugcugucgacaugauccaga	3	0	JCK-3
agugcugucgacaugauccagU	1	1	LT-3
Ggugcugucgacaugauccaga	14	1	JCK-1
agugcugucgacaugauccaga	29	0	LT-2
agugcugucgacaugauccaga	8	0	JCK-1
agNgcugucgacaugauccaga	1	1	LT-2
agugUugucgacaugauccaga	1	1	LCK-3
Ggugcugucgacaugauccaga	18	1	JCK-2
agugcugucgacaAgauccaga	1	1	JCK-3
Ggugcugucgacaugauccaga	13	1	JCK-3
agugcugucgacaAgauccaga	5	1	JCK-1
agugcugucgacaugauccaga	35	0	LT-1
agugcugucgacaugauccaga	16	0	LCK-3
agugcugucgacaAgauccagaa	1	1	JCK-2
agugcugucgacaugauccagaag	1	0	JCK-2
gugcugucgacaugaucc	44	0	LT-2
gugcugucgacaAgaucc	18	1	JCK-3
gugcugucgacaugaucc	16	0	LCK-2
gugcugucgaGaugaucc	1	1	JCK-1
gugcugucgacaAgaucc	16	1	JCK-1
gugcugucgacaugaucc	100	0	JCK-1
gugcugucgaAaugaucc	1	1	JCK-2
gugcugucgacaugaucc	29	0	LT-1
gugcugucgCcaugaucc	1	1	JCK-2
gugcugucgacaugaucc	107	0	JCK-3
gugcugucgacaAgaucc	42	1	JCK-2
gugcugucgacaugaucc	14	0	JT-2
gugcugucgacaAgaucc	5	1	JT-3
Augcugucgacaugaucc	1	1	JCK-1
gugcugucgacaugaucc	24	0	LCK-3
gugcugucgacaugaucc	148	0	JCK-2
gugcugucgacaugaucc	54	0	JT-1
gugcugucgacaAgaucc	3	1	JT-2
gugcugucgacaugaucc	63	0	LT-3
gugcugucgacaugaucc	12	0	JT-3
gugcugucgacaugaucc	17	0	LCK-1
gugcugucgacaugCucca	1	1	JCK-3
gugcugucgacaugaucca	29	0	JCK-1
gugcugucgacGugaucca	1	1	JT-1
gugcugucgacaAgaucca	7	1	JT-1
gugcugucgacaugaucca	32	0	LT-2
gugcugucgacaAgaucca	27	1	JCK-2
gugcugucgacaAgaucca	7	1	JT-2
gugcugucgacaugaucca	37	0	JT-1
gugcugucgacaugaucca	31	0	LT-3
gugcugucgacaugaucca	29	0	LCK-3
gNgcugucgacaugaucca	1	1	LCK-1
gugcugucgacaugNucca	1	1	LCK-1
gugcugucgacaugaucca	31	0	JT-2
gugcugucgacaAgaucca	11	1	JCK-1
gugcugucgacaAgaucca	8	1	JT-3
gugcugucgacaugaucca	50	0	JCK-2
guNcugucgacaugaucca	1	1	LT-2
gugcugucgacaugaucca	25	0	LCK-2
gugcugucgacaugaucca	28	0	JCK-3
gugcugucgacaugGucca	1	1	JT-1
gugcugucgacaAgaucca	9	1	JCK-3
gugcugucgacaugaucca	25	0	JT-3
gugcugucgacaugaucca	19	0	LCK-1
gugcugucgacaugaucca	21	0	LT-1
gugcugucgacaugauccag	4	0	JT-1
Augcugucgacaugauccag	1	1	JCK-2
gugcugucgacaugauccaA	2	1	JCK-1
gugcugucgacaugauccag	62	0	LT-3
gugcugucgacaAgauccag	11	1	JCK-3
gugcugucgacaAgauccag	9	1	JT-3
gugcugucgacaugauccag	84	0	LCK-3

Mature | Star

uguuuucaucaacaguuagguuaaaaagaaaaugagcuccucuuauuuugucaucaaguauaaaaugacggcgagcucuuacagaugaaagcagugcgguuccaucuuguucguuuacccucuggaucuuguucgacagcacucuccuauauuuugccauuuaucuuuaaaugcgaaaucuuuaaaugcaaaauggcgacgauagagagugcugucgacaugauccagaaggugcagucgaauaagau

gugcugucgacaAgauccag	32	1	JCK-2
gugcugucgacaugauccCg	1	1	JCK-2
gugcugucgacaAgauccag	9	1	JT-2
gugcugucgacaugauccaA	1	1	LT-3
gugcugucgacaugauccaA	1	1	LT-2
gugcugucgacaugauccag	27	0	LT-2
gugcugucgacaugauccaA	1	1	JCK-3
gugcugucgacaugauccag	4	0	JT-2
gugcugucgacaugauccag	39	0	LT-1
gugUugucgacaugauccag	1	1	LCK-3
gugcugucgacaAgauccag	7	1	JCK-1
gugcugucgacaugauccag	110	0	JCK-2
gugcugucgacaugauccag	54	0	JCK-1
gugcugucgacaugauccag	84	0	JCK-3
gugcugucgacaugauccag	10	0	JT-3
gugcugucgCcaugauccaga	1	1	LT-1
gugcugucgacaugauccaga	464	0	LT-1
gugcugucgacaugCuccaga	2	1	JCK-1
gugcugucgGcaugauccaga	1	1	JCK-3
gugcugucgacaugauccaUa	3	1	JCK-2
gugcugucgacaugNuccaga	1	1	LCK-1
gugcugucgacaugauccaga	2107	0	JCK-1
gNgcugucgacaugauccaga	3	1	JCK-3
gugcugucgacCugauccaga	2	1	JCK-3
gugcugucgacaugaGccaga	1	1	JCK-2
gNgcugucgacaugauccaga	1	1	JCK-2
gugcugucgacaAgauccaga	1129	1	JCK-2
gugcugucgacaugaucUaga	4	1	JCK-2
gugcugucgacCugauccaga	4	1	JCK-1
gugcugucgacCugauccaga	2	1	LT-2
gugcugucgacaugauccaga	48	0	JT-2
gugcugucgacaugauccagC	1	1	LT-3
gugUugucgacaugauccaga	1	1	LT-3
gugcugucgaAaugauccaga	1	1	JCK-3
gAgcugucgacaugauccaga	2	1	JCK-2
Cugcugucgacaugauccaga	1	1	LT-3
gugcugucgaGaugauccaga	1	1	JCK-1
gugcugucgacaugauccCga	3	1	JCK-1
gugcugucgacaugauccCga	2	1	JCK-2
gugcugucgacCugauccaga	1	1	LCK-2
gugUugucgacaugauccaga	9	1	JCK-3
Augcugucgacaugauccaga	5	1	JCK-2
gugcugucgacauAauccaga	1	1	JCK-2
guNcugucgacaugauccaga	2	1	LT-2
gugcugucgCcaugauccaga	2	1	LT-2
gCgcugucgacaugauccaga	1	1	JCK-2
Augcugucgacaugauccaga	1	1	JCK-1
gugcugucgacaAgauccaga	910	1	JCK-3
gugcugucgacaAgauccaga	22	1	JT-3
gugcugucgacaugauccaUa	2	1	JCK-3
gugcugucgacaugauccaAa	4	1	JCK-2
gugcugucgacaCgauccaga	1	1	JCK-3
gugcugucgacaugauccaga	74	0	JT-3
gugcugucgacaugaucUaga	2	1	JCK-1
gugcugucgacaugCuccaga	2	1	JCK-3
gugcugucgCcaugauccaga	3	1	JCK-2
gugcugucgacaugaucGaga	1	1	JCK-1
Augcugucgacaugauccaga	1	1	LT-3
gugcugucgacaugCuccaga	2	1	LT-3
gugcugucgacCugauccaga	1	1	LCK-1
gugcugucgacaugCuccaga	2	1	JCK-2
gugcugucgacaugauccaga	315	0	LCK-3
gugcugucgacaugauccGga	1	1	LT-3
Augcugucgacaugauccaga	2	1	LCK-3
gugcugucgacaugauccagN	1	1	JCK-1
gAgcugucgacaugauccaga	1	1	JCK-3
gugcugucgCcaugauccaga	2	1	JCK-3
gugcugucgacaugauccaga	34	0	LCK-1
Uugcugucgacaugauccaga	2	1	JCK-2
gugcuguUgacaugauccaga	1	1	JCK-2

Mature Star

```
uguuuucaucaacaguuagguuaaaaagaaaaugagcuccucuuauuuugucaucaaguauaaaaugacggcgagcucuuacagaugaaagcagugcgguuccaucuuguucguuuacccucuggaucuuguucgacagcacucuccuauauuuugccauuuaucuuuaaaugcgaaaucuuuaaaugcaaaauggcgacgaugauaggagugcugucgacaugauccagaaggugcagucgaauaagau
..................................................................................................................................................................................................................gugcugucgacaAgauccaga...................   712    1    JCK-1
..................................................................................................................................................................................................................gugcugucgacaugauccaga...................   2674   0    JCK-3
..................................................................................................................................................................................................................gugcugucgCcaugauccaga...................   6      1    JCK-1
..................................................................................................................................................................................................................gugcugucgacaAgauccaga...................   18     1    JT-2
..................................................................................................................................................................................................................gugUugucgacaugauccaga...................   9      1    JCK-2
..................................................................................................................................................................................................................gugcugucgacaugauccaNa...................   1      1    JCK-1
..................................................................................................................................................................................................................Augcugucgacaugauccaga...................   8      1    JCK-3
..................................................................................................................................................................................................................gugcugucgacCugauccaga...................   1      1    JCK-2
..................................................................................................................................................................................................................guUcugucgacaugauccaga...................   2      1    JCK-3
..................................................................................................................................................................................................................gugcugucNacaugauccaga...................   1      1    JCK-3
..................................................................................................................................................................................................................gugcugucgacaugauccGga...................   2      1    JCK-1
..................................................................................................................................................................................................................gugcugucgacaAgauccaga...................   19     1    JT-1
..................................................................................................................................................................................................................gugNugucgacaugauccaga...................   3      1    JCK-1
..................................................................................................................................................................................................................gugcugucgacaugauccagG...................   3      1    LCK-3
..................................................................................................................................................................................................................Cugcugucgacaugauccaga...................   1      1    LCK-3
..................................................................................................................................................................................................................gAgcugucgacaugauccaga...................   1      1    JCK-1
..................................................................................................................................................................................................................gugcugucgacaugauccaga...................   48     0    LCK-2
..................................................................................................................................................................................................................Cugcugucgacaugauccaga...................   1      1    JCK-1
..................................................................................................................................................................................................................gugGugucgacaugauccaga...................   1      1    JCK-1
..................................................................................................................................................................................................................gugcugucgacaugauccaga...................   23     0    JT-1
..................................................................................................................................................................................................................Augcugucgacaugauccaga...................   1      1    LT-1
..................................................................................................................................................................................................................guAcugucgacaugauccaga...................   2      1    LT-2
..................................................................................................................................................................................................................gugcugucgacaugauccagU...................   2      1    JCK-3
..................................................................................................................................................................................................................gugcugucgacCugauccaga...................   2      1    LT-1
..................................................................................................................................................................................................................gugUugucgacaugauccaga...................   8      1    JCK-1
..................................................................................................................................................................................................................Uugcugucgacaugauccaga...................   1      1    LT-2
..................................................................................................................................................................................................................gugcugucgaAaugauccaga...................   1      1    LT-2
..................................................................................................................................................................................................................gugcugucgacaugaucUaga...................   1      1    JCK-3
..................................................................................................................................................................................................................gugcugucgacaCgauccaga...................   1      1    JCK-2
..................................................................................................................................................................................................................gugcugucgacaugauccaga...................   471    0    LT-2
..................................................................................................................................................................................................................Augcugucgacaugauccaga...................   1      1    LT-2
..................................................................................................................................................................................................................gugcugucgacaugauccagU...................   1      1    JCK-2
..................................................................................................................................................................................................................gugUugucgacaugauccaga...................   2      1    LT-1
..................................................................................................................................................................................................................gugcugucgacaugauccaga...................   3681   0    JCK-2
..................................................................................................................................................................................................................gugcugucgacNugauccaga...................   5      1    JCK-1
..................................................................................................................................................................................................................gNgcugucgacaugauccaga...................   3      1    JCK-1
..................................................................................................................................................................................................................gugcugucgacaugaGccaga...................   1      1    JCK-1
..................................................................................................................................................................................................................gugcugucgaAaugauccaga...................   1      1    JCK-1
..................................................................................................................................................................................................................guAcugucgacaugauccaga...................   1      1    JCK-3
..................................................................................................................................................................................................................gugcugucgacaugauccCga...................   1      1    LT-3
..................................................................................................................................................................................................................gugcugucgacaugauccaga...................   686    0    LT-3
..................................................................................................................................................................................................................gugcugucgacaugauccagaG..................   1      1    LT-1
..................................................................................................................................................................................................................gugcugucgacaugauccagaa..................   3      0    LT-2
..................................................................................................................................................................................................................gugcugucAacaugauccagaa..................   5      1    JCK-2
..................................................................................................................................................................................................................gugcugucgacaAgauccagaa..................   6      1    JCK-1
..................................................................................................................................................................................................................gugcugucgacaugauccagaa..................   6      0    JCK-3
..................................................................................................................................................................................................................gugcugucgacaugauccagaa..................   11     0    JCK-2
..................................................................................................................................................................................................................gugcugucgacaugauccagaa..................   4      0    JCK-1
..................................................................................................................................................................................................................gugcugucgacaAgauccagaa..................   2      1    JCK-3
..................................................................................................................................................................................................................gugcugucgacaAgauccagaa..................   7      1    JCK-2
..................................................................................................................................................................................................................gugcugucgacaugauccagaaU.................   2      1    JCK-2
..................................................................................................................................................................................................................gugcugucgacaugauccagaaA.................   1      1    JCK-1
..................................................................................................................................................................................................................gugcugucgacaugauccagaaU.................   1      1    JCK-1
..................................................................................................................................................................................................................gugcugucgacaugauccagaCg.................   1      1    LT-1
..................................................................................................................................................................................................................gugcugucgacaugauccagaagg................   2      0    LT-1
..................................................................................................................................................................................................................gugcugucgacaugauccagaaggu...............   2      0    JCK-2
...................................................................................................................................................................................................................ugcugucgacaugaucca.....................   1      0    LT-1
...................................................................................................................................................................................................................ugcugucgacaugaucca.....................   2      0    LCK-3
...................................................................................................................................................................................................................ugcugucgacaugaucca.....................   1      0    JCK-2
...................................................................................................................................................................................................................ugcugucgacaugaucca.....................   6      0    LT-3
...................................................................................................................................................................................................................ugcugucgacaugaucca.....................   5      0    LT-2
...................................................................................................................................................................................................................ugcugucgacaugauccaA....................   1      1    JCK-2
...................................................................................................................................................................................................................ugcugucgacaugauccag....................   2      0    JCK-2
...................................................................................................................................................................................................................ugcugucgacaugauccag....................   1      0    JCK-3
...................................................................................................................................................................................................................ugcugucgacaugauccag....................   2      0    LT-3
...................................................................................................................................................................................................................ugcugucgacaugauccaC....................   1      1    JCK-1
...................................................................................................................................................................................................................ugcugucgacaAgauccag....................   2      1    JCK-3
...................................................................................................................................................................................................................ugcugucgacaugauccaA....................   2      1    JCK-1
...................................................................................................................................................................................................................ugcugucgacaAgauccag....................   2      1    JCK-1
...................................................................................................................................................................................................................ugcugucgacaAgauccag....................   2      1    JCK-2
```

```
                                                                                                                          Mature                                                                                      Star
uguuuucaucaacaguuagguuaaaaagaaaaugagcuccucuuauuuugucaucaaguauaaaaugacggcgagcucuuacagaugaaagcagugcgguuccaucuuguucguuuacccucuggaucuuguucgacagcacucuccuauauuuugccauuuaucuuuaaaugcgaaaucuuuaaaugcaaaauggcgacgaugauaggagugcugucgacaugauccagaaggugcagucgaauaagau
...................................................................................................................................................................................................................uNcugucgacaugauccaga...................   1     1   JCK-2
...................................................................................................................................................................................................................ugcugucgacaugauccaga...................   17    0   LCK-2
...................................................................................................................................................................................................................ugcugucgacaugaucUaga...................   1     1   JCK-3
...................................................................................................................................................................................................................ugcugucgacaugauccaga...................   51    0   JCK-2
...................................................................................................................................................................................................................ugcugucgacaAgauccaga...................   22    1   JT-1
...................................................................................................................................................................................................................ugcugucgacaugauccaga...................   1     0   JT-3
...................................................................................................................................................................................................................ugcugucgacaugauccaga...................   10    0   JT-1
...................................................................................................................................................................................................................ugcugucgacaAgauccaga...................   125   1   JCK-2
...................................................................................................................................................................................................................ugcugucgacaAgauccaga...................   105   1   JCK-3
...................................................................................................................................................................................................................ugcugucgacaugauccaga...................   25    0   JCK-1
...................................................................................................................................................................................................................ugcugucgacaAgauccaga...................   79    1   JCK-1
...................................................................................................................................................................................................................ugcugucgacaAgauccaga...................   13    1   JT-2
...................................................................................................................................................................................................................ugcugucgacaugauccaga...................   20    0   LT-3
...................................................................................................................................................................................................................ugUugucgacaugauccaga...................   1     1   JCK-2
...................................................................................................................................................................................................................ugcugucgacaugauccaga...................   24    0   LCK-1
...................................................................................................................................................................................................................ugcugucgacaugauccaga...................   29    0   JCK-3
...................................................................................................................................................................................................................ugcugucgacaugauccaga...................   20    0   LCK-3
...................................................................................................................................................................................................................ugcugucgacaAgauccaga...................   20    1   JT-3
...................................................................................................................................................................................................................ugcugucgacaugauccaga...................   15    0   LT-1
...................................................................................................................................................................................................................ugcugucgacaugauccaga...................   29    0   LT-2
...................................................................................................................................................................................................................ugcugucgacaugauccagaC..................   1     1   JCK-2
...................................................................................................................................................................................................................ugcugucgacaugauccagaU..................   1     1   JCK-3
...................................................................................................................................................................................................................ugcugucgacaAgauccagaa..................   1     1   JCK-3
...................................................................................................................................................................................................................ugcugucgacaugauccagaa..................   1     0   JCK-2
...................................................................................................................................................................................................................ugcugucgacaugauccagaC..................   2     1   LT-1
...................................................................................................................................................................................................................ugcugucgacaAgauccagaa..................   2     1   JCK-1
...................................................................................................................................................................................................................ugcugucgacaugauccagaag.................   1     0   LT-3
...................................................................................................................................................................................................................ugcugucgacaugauccagaag.................   1     0   LT-1
...................................................................................................................................................................................................................ugcugucgacaAgauccagaaggu...............   1     1   JCK-2
...................................................................................................................................................................................................................ugcugucgacaugauccagaaggu...............   1     0   JCK-3
....................................................................................................................................................................................................................gcugucgacaAgauccagaag.................   1     1   JCK-2
....................................................................................................................................................................................................................gcugucgacaAgauccagaag.................   3     1   JCK-3
....................................................................................................................................................................................................................gcugucgacaugauccagaag.................   1     0   LT-1
....................................................................................................................................................................................................................gcugucgacaAgauccagaag.................   3     1   JT-2
....................................................................................................................................................................................................................gcugucgacaugauccagaag.................   2     0   JCK-2
....................................................................................................................................................................................................................gcugucgacaAgauccagaag.................   4     1   JT-3
....................................................................................................................................................................................................................gcugucgacaAgauccagaag.................   3     1   JCK-1
.....................................................................................................................................................................................................................cugucgacaugauccaga...................   2     0   JT-3
.....................................................................................................................................................................................................................cugucgacaAgauccaga...................   1     1   JCK-1
.....................................................................................................................................................................................................................cugucgacaugauccaga...................   1     0   JT-2
.....................................................................................................................................................................................................................cugucgacaugauccaga...................   2     0   LT-2
.....................................................................................................................................................................................................................cugucgacaugauccaga...................   1     0   JCK-1
.....................................................................................................................................................................................................................cugucgacaugauccaga...................   1     0   JCK-3
.....................................................................................................................................................................................................................cugucgacaAgauccaga...................   1     1   JCK-2
.....................................................................................................................................................................................................................cugucgacaugauccaga...................   1     0   LT-3
.....................................................................................................................................................................................................................cugucgacaugauccaga...................   1     0   LT-1
.....................................................................................................................................................................................................................cugucgacaugauccagaa..................   1     0   LT-2
.....................................................................................................................................................................................................................cugucgacaugCuccagaa..................   1     1   JCK-2
.....................................................................................................................................................................................................................cugucgacaugauccagaa..................   6     0   LCK-3
.....................................................................................................................................................................................................................cugucgacaAgauccagaa..................   2     1   JCK-2
.....................................................................................................................................................................................................................cugucgacaugauccagaa..................   1     0   JCK-2
.....................................................................................................................................................................................................................cugucgacaugauccagaa..................   1     0   JCK-1
.....................................................................................................................................................................................................................cugucgacaAgauccagaa..................   2     1   JCK-1
.....................................................................................................................................................................................................................cugucgacaugauccagaa..................   1     0   LT-1
.....................................................................................................................................................................................................................cugucgacaAgauccagaa..................   1     1   JCK-3
.....................................................................................................................................................................................................................cugucgacaugauccagaag.................   2     0   JCK-3
.....................................................................................................................................................................................................................cugucgacaAgauccagaag.................   5     1   JCK-3
.....................................................................................................................................................................................................................cugucgacaugauccagaag.................   1     0   LT-3
.....................................................................................................................................................................................................................cugucgacaAgauccagaag.................   1     1   JCK-1
.....................................................................................................................................................................................................................cugucgacaugauccagaaU.................   1     1   LT-3
.....................................................................................................................................................................................................................cugucgacaugauccagaag.................   5     0   JCK-1
.....................................................................................................................................................................................................................cugucgacaugauccagaag.................   6     0   JCK-2
.....................................................................................................................................................................................................................cugucgacaugauccagaag.................   5     0   LT-1
.....................................................................................................................................................................................................................cugucgacaAgauccagaag.................   4     1   JCK-2
.....................................................................................................................................................................................................................cugucgacaAgauccagaagg................   3     1   JCK-3
.....................................................................................................................................................................................................................cugucgacaugauccagaagU................   3     1   JCK-1
.....................................................................................................................................................................................................................cugucgacaugauccagaagg................   1     0   JCK-2
.....................................................................................................................................................................................................................cugucgacaugauccagaagg................   1     0   JCK-3
.....................................................................................................................................................................................................................cugucgacaAgauccagaagg................   5     1   JCK-1
.....................................................................................................................................................................................................................cugucgacaAgauccagaagg................   4     1   JCK-2
```

Mature Star

```
uguuuucaucaacaguuagguuaaaaagaaaaugagcuccucuuauuuugucaucaaguauaaaaugacggcgagcucuuacagaugaaagcagugcgguuccaucuuguucguuuacccucuggaucuuguucgacagcacucuccuauauuuugccauuuaucuuuaaaugcgaaaucuuuaaaugcaaaauggcgacgaugauaggagugcugucgacaugauccagaaggugcagucgaauaagau
.....................................................................................................................................................................................................................cugucgacaugauccagaagU................	1	1	LT-3
......................................................................................................................................................................................................................ugucgacaAgauccagaagg................	1	1	JCK-1
......................................................................................................................................................................................................................ugucgacaAgauccagaaggu...............	11	1	JCK-2
......................................................................................................................................................................................................................ugucgacaugauccagaaggu...............	1	0	LT-2
......................................................................................................................................................................................................................ugucgacaAgauccagaaggu...............	6	1	JCK-1
......................................................................................................................................................................................................................ugucgacaAgauccagaaggu...............	17	1	JCK-3
........................................................................................................................................................................................................................ucgacaAgauccagaaggugc.............	2	1	JCK-1
........................................................................................................................................................................................................................ucgacaAgauccagaaggugc.............	3	1	JCK-3
........................................................................................................................................................................................................................ucgacaAgauccagaaggugc.............	1	1	JCK-2
```
